# Silver Cluster Interactions with Tyrosine: Towards Amino Acid Detection

**DOI:** 10.3390/ijms23020634

**Published:** 2022-01-06

**Authors:** Andrey A. Buglak, Alexei I. Kononov

**Affiliations:** The Faculty of Physics, Saint Petersburg State University, 199034 St. Petersburg, Russia; a.kononov@spbu.ru

**Keywords:** tyrosine, silver nanoclusters, aromatic amino acid detection, amino acid complexes with metals, density functional theory, SERS

## Abstract

Tyrosine (Tyr) is involved in the synthesis of neurotransmitters, catecholamines, thyroid hormones, etc. Multiple pathologies are associated with impaired Tyr metabolism. Silver nanoclusters (Ag NCs) can be applied for colorimetric, fluorescent, and surface-enhanced Raman spectroscopy (SERS) detection of Tyr. However, one should understand the theoretical basics of interactions between Tyr and Ag NCs. Thereby, we calculated the binding energy (E_b_) between Tyr and Ag_n_^q^ (n = 1–8; q = 0–2) NCs using the density functional theory (DFT) to find the most stable complexes. Since Ag NCs are synthesized on Tyr in an aqueous solution at pH 12.5, we studied Tyr^−1^, semiquinone (SemiQ^−1^), and Tyr^−2^. Ag_3_^2+^ and Ag_5_^+^ had the highest E_b_. The absorption spectrum of Tyr^−2^ significantly red-shifts with the attachment of Ag_3_^2+^, which is prospective for colorimetric Tyr detection. Ag_3_^2+^ interacts with all functional groups of SemiQ^−1^ (phenolate, amino group, and carboxylate), which makes detection of Tyr possible due to band emergence at 1324 cm^−1^ in the vibrational spectrum. The ground state charge transfer between Ag and carboxylate determines the band emergence at 1661 cm^−1^ in the Raman spectrum of the SemiQ^−1^–Ag_3_^2+^ complex. Thus, the prospects of Tyr detection using silver nanoclusters were demonstrated.

## 1. Introduction

Subnanometer metal nanoclusters (NCs) have gained increasing attention in the last decade due to their applicability in biocatalysis, bioimaging, and biosensing. Noble metal NCs possess high biocompatibility, chemical stability, low toxicity, and low photobleaching [1]. This set of properties allows them to be used both in vitro and in vivo. Obviously, it is possible to use Ag NCs for biosensor determination of Tyr in aqueous solutions by colorimetric, luminescent, and surface-enhanced Raman spectroscopy (SERS) methods.

Thus, absorption spectra of the ligand and NC are transformed upon the attachment of metal NCs, which can be used for detection of the complex [2]. The same is true for fluorescence spectra [3,4,5]. When interacting with metals, vibrational and Raman spectra undergo certain changes, usually called chemical enhancement, which is exploited by SERS [6]. These types of detection are widely exploited by biosensors [2,7]. Biosensing surpasses more traditional methods, for example, high-performance liquid chromatography (HPLC), infrared spectroscopy, and bioluminescence, because of its higher selectivity, accuracy, and cost-effectiveness [8,9]. Biosensors for various analytes are in great demand nowadays. Biosensors exploit the unique properties of metal nanoclusters and nanoparticles: sensitivity, chemical stability, low toxicity, low photobleaching, intense luminescence, etc [10].

**Figure 1:**
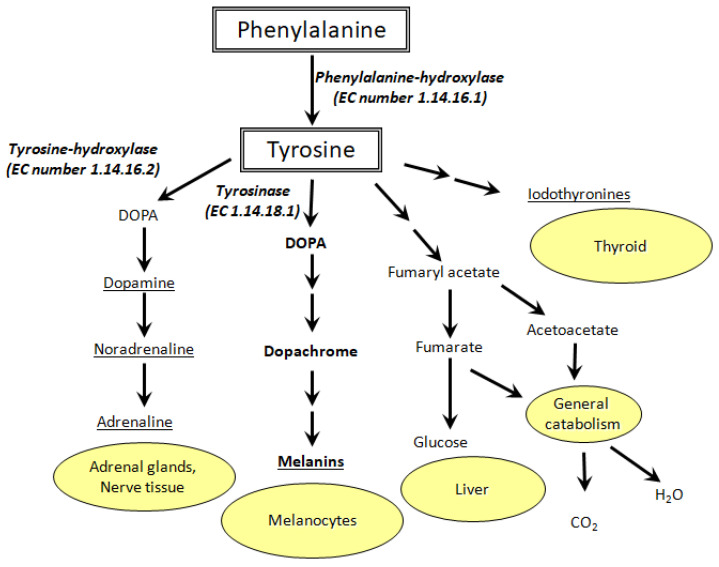
Metabolic pathways of tyrosine oxidation and their biological role (EC stands for enzyme commission).

Tyrosine is formed in vivo through hydroxylation of phenylalanine by the phenylalanine-hydroxylase (PAH) enzyme. The significance of tyrosine biosynthesis is huge since it is one of 20 α-proteinogenic amino acids. Tyrosine is also involved in biosynthesis of DOPA, neurotransmitters, catecholamines (dopamine, adrenaline, norepinephrine), thyroid hormones, melanins, etc. (Figure 1) [11,12]. There are a number of pathologies associated with impaired tyrosine metabolism: phenylketonuria, hypothyroidism, tyrosinemia, alkaptonuria, and vitiligo [13]. This means that precise determination of Tyr concentration in biological fluids is extremely important [14].

Vitiligo, for example, is characterized by the appearance of depigmented skin patches. Due to the lack of tyrosinase activity and low 3,4-dioxophenylalanine (DOPA) concentration in melanocytes, melanin synthesis can be disrupted [15]. The etiology of vitiligo is still unknown, but its relationship with the metabolism of aromatic amino acids (Phe, Tyr, DOPA, Trp) is known for certain [16]. Redox reactions of Tyr and DOPA [17,18], as well as their interplay with pterin photoreactions [19,20,21,22,23] are of great importance for melanogenesis and vitiligo etiology/treatment [24,25,26,27,28]. In this regard, biosensors based on metal NCs can be used for cost-effective diagnostics of vitiligo and other Tyr metabolism-related diseases. In particular, silver NCs can help to detect tyrosine in biological fluids and cellular homogenates with high selectivity and sensitivity. We suppose that a cluster should interact with all functional groups of Tyr: amine, carboxyl, and phenol.

Usually, metal NCs are synthesized in solution on biopolymer templates: nucleic acids or proteins [3,29,30]. However, the synthesis is also possible on low molecular weight compounds: thiolates, organophosphates, and amino acids [31]. As is known, Tyr is able to reduce Ag^+^ [32]. We have demonstrated that twice deprotonated tyrosine (Tyr^−2^) can serve as a template for silver NC synthesis in an aqueous solution with an alkaline pH even without the addition of any reducing agent [33]. In this regard, the ability of silver NCs to be synthesized on Tyr can be used for the development of new Ag NC-based Tyr biosensors.

Theoretical studies of amino acid–Ag interactions are not as numerous as they should be. Investigations of silver interactions with histidine [34], glycine, and cysteine [35] were made by Zahra Jamshidi’s group; this research mainly concerned three atomic Ag cluster complexes. Roland Mitric’s group conducted a study of silver NC interactions with tryptophan and histidine [36,37]. Our previous studies were mostly about diatomic Ag clusters and a complete set of amino acids [38,39]. Thus, there is a lack of systematic theoretical studies of amino acid interactions with silver clusters of different sizes and charges. Only a theoretical study of cysteine interactions with silver NCs *n* = 2–10 has been published recently [40].

In this work, we have studied Tyr interactions with Ag_n_^q^ clusters in a wide range of sizes and charges (*n* = 1–8, q = 0–2) NCs. We have evaluated the binding energy, found the most stable Tyr–Ag complexes, established vibrational and electronic UV-vis spectra, and outlined the prospects of experimental Tyr detection.

## 2. Computational Details

The geometries have been obtained for silver nanoclusters Ag_n_^q^ (*n* = 1–8, q = 0–2). For each isolated cluster, starting from *n* = 4, at least 4 isomers have been regarded to have the most stable geometry, both free and in complex with Tyr. Prior to geometry optimization, we have performed a conformational analysis; we have searched for the most favorable conformation of Tyr in each complex and the most favorable site of Ag NC attachment. Next, we have performed geometry optimizations and calculated the binding energy between Tyr and NC.

Geometry optimization and binding energy calculation have been done with density functional theory (DFT) at the PBE level [41] with the Grimme D3 dispersion correction [42] using the Orca 3.0 program package [43]. The Pople basis set 6-31G(d,p) has been used, and Ag atoms have been treated with LANLTZ effective core potential (ECP) [44]. The COSMO solvation model [45] has been used to take water into account. The initial geometries of the Tyr–Ag_n_^q^ complexes have been constructed by placing Ag NCs near the active sites of Tyr: N and O. N and O are electron-rich atoms, which can donate electron density to silver from their lone electron pairs.

Binding energies (E_b_) have been calculated with PBE-D3 for the reaction:

Tyr + Ag_n_^q^ → Tyr–Ag_n_^q^

The equation for the binding energy E_b_ is the following:

E_b_ = E_Tyr-Agn_ − (E_Tyr_ + E_Agn_)

Bader’s quantum theory “Atoms-In-Molecules” (QTAIM) [46] analysis has been performed using the Multiwfn program package [47]. Electron densities of Moller-Plesset perturbation theory MP2 calculations have been applied to evaluate the properties of bond critical points (BCPs), as in our previous paper [38].

We have calculated UV-vis absorption spectra for the most energetically favorable complexes. Since MP2 gives better geometry predictions for ligand-protected Ag NCs than DFT [48], we have established the MP2 geometries prior to absorption spectra calculation. The MP2 realized in Orca 3.0 has been used along with the 6-31G(d,p) basis set and LANLTZ ECP as before [38]. Electronic absorption spectra have been obtained using the time-dependent density functional theory (TDDFT), namely the M062X functional [49] with the def2-TZVP basis set and def2-TZVP ECP for silver [50]. The M062X/def2-TZVP approach has shown good accuracy in predicting the absorption spectra of ligand-protected small silver clusters as compared to superior ab initio methods [51].

Vibrational and Raman spectra of the Tyr–Ag complexes and the isolated Tyr have been obtained using DFT: the B3LYP functional with D3 dispersion correction, the 6-31G(d,p) basis set, LANLTZ ECP, and the COSMO model for water. The B3LYP shows good accuracy in predicting vibrational [52] and Raman [53,54] spectra as compared to other DFT functionals and even the MP2 method. The natural bond orbital (NBO) analysis has been done using the B3LYP in Gaussian 16 [55].

The stabilization energy E^(2)^ of the *i* → *j* delocalization has been calculated for each donor NBO orbital *i* and acceptor orbital *j*, according to the following equation:

(1) $$E^{\left(2\right)}=-2\frac{\langle i\left|\hat{F}\right|{j\rangle }^{2}}{e_{j}-e_{i}},$$

where $e_{i}$ and $e_{j}$ are the NBO orbital energies, and $\hat{F}$ is the Fock operator.

The amount of charge transferred from the *i* orbital to the *j* orbital has also been calculated using the Fock operator and the NBO orbital energies in accordance with Equation (2):

(2) $$q_{\mathrm{CT}}=2{\left(\frac{\langle i\left|\hat{F}\right|j\rangle }{e_{j}-e_{i}}\right)}^{2}$$

## 3. Results and Discussion

### 3.1. Isolated Silver Nanoclusters

First, we analyzed isolated Ag_n_^q^ (*n* = 1–8, q = 0–2) nanoclusters. Ag_2_^2+^ has not been realized due to Coulomb repulsion. The Ag–Ag distance is equal to 2.59 Å and 2.72 Å for Ag_2_^0^ and Ag_2_^+^, respectively, according to PBE-D3.

**Figure 2:**
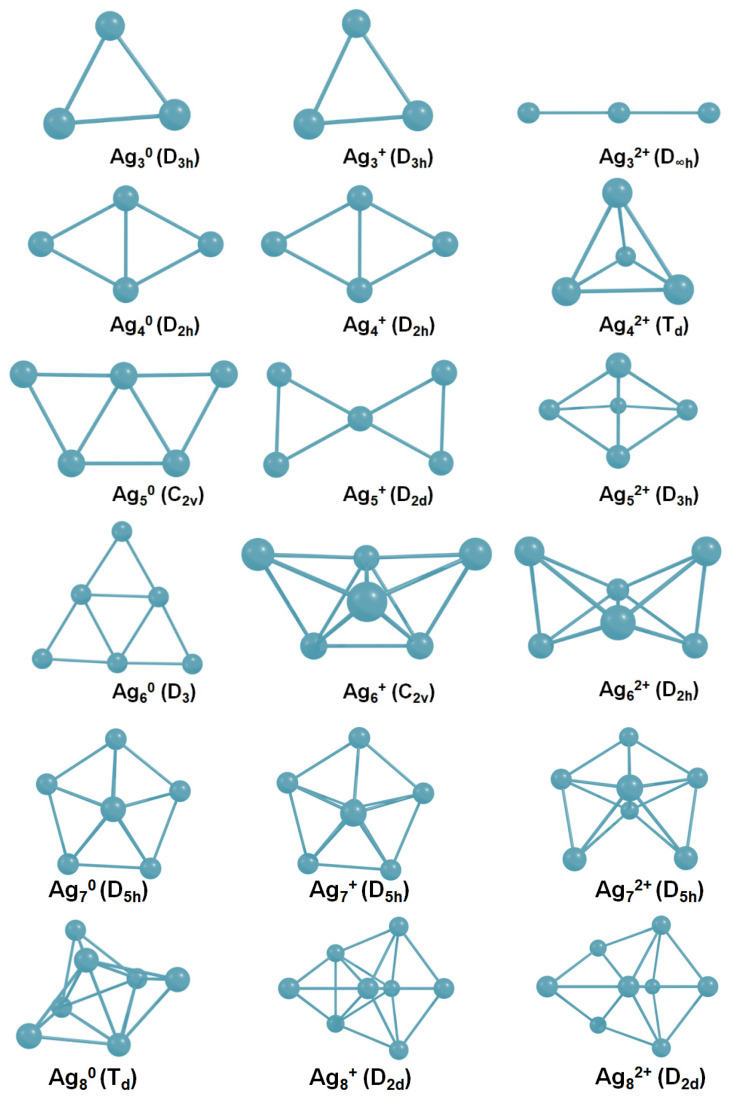
The most stable isomers for isolated Ag_n_^q^ (*n* = 3–8, q = 0–2) nanoclusters.

Ag_3_^q^ (q = 0, +1, +2) clusters have two types of geometry: linear (D_∞h_ symmetry) and triangular (D_3h_). The triangle is the most stable system for q = 0–1, whereas the linear structure is the most energetically favorable for q = +2 (Figure 2). Two bonds of the Ag_3_^0^ NC are 2.67 Å, whereas the third bond equals 2.96 Å. Each bond of the Ag_3_^+^ cluster is 2.7 Å. Ag_3_^2+^ possesses bond lengths equal to 2.84 Å. It should be mentioned that the Ag–Ag bond distance increases with the growth of cluster charge, which is observed for all the clusters with *n* = 2–8.

Two geometries are the most feasible for the isolated Ag_4_^q^ (q = 0–2) clusters: D_2h_ diamond (Ag_4_^0^ and Ag_4_^+^) and T_d_ tetrahedron (Ag_4_^2+^).

Free Ag_5_ clusters have different structures depending on the charge. C_2v_ plane trapezoid is the most favorable form of Ag_5_^0^. Ag_5_^+^ is also characterized by plane 2D geometry (D_2d_). However, the Ag_5_^2+^ dicationic cluster possesses a 3D trigonal bipyramid D_3h_ structure (Figure 2).

Ag_6_ clusters also have different geometries depending on the charge. Isolated Ag_6_^0^ is a 2D system with a triangular shape (D_3_). The most stable isomer of Ag_6_^+^ is a 3D tetragon pair with a shared bond and an edge (C_2v_ symmetry). The most energetically favorable geometry of Ag_6_^2+^ is three-dimensional (D_2h_).

All Ag_7_ NCs possess the same D_2h_ symmetry (Figure 2). Apparently, this pentagonal bipyramid structure is very stable for silver NCs, and even the charge does not play a significant role in this case.

Neutral and positively charged Ag_8_ NCs have different geometries. The Ag_8_^0^ cluster, which is often called a magical system [56], possesses T_d_ geometry. Both Ag_8_^+^ and Ag_8_^2+^ NCs have D_2d_ symmetry.

Therefore, the structure of isolated silver NCs strongly differs depending on the cluster charge. Presumably, clusters can change geometry upon interaction with Tyr. Isolated neutral silver clusters remain two-dimensional with up to six atoms. Ag_n_^+^ clusters are also subjected to 2D–3D transformation at *n* = 6. Dicationic Ag_n_^2+^ clusters transform from linear to three-dimensional geometry, starting from *n* = 4. The established results are in agreement with previously reported studies on isolated silver NCs, both theoretical and experimental [57,58,59].

### 3.2. Interaction of Ag NCs with Tyrosine

We have performed calculations for the Tyr complexes with silver NCs Ag_n_^q^ (*n* = 1–8, q = 0–2). Previously, we have shown that silver NCs are most effectively synthesized on tyrosine in an aqueous solution at pH 12.5 [33], which means that the synthesis occurs with the participation of twice deprotonated tyrosine Tyr^−2^. It is also known that tyrosine can reduce Ag^+^, forming a semiquinone (SemiQ) [32]. In this regard, the calculations have been done for three forms of tyrosine: Tyr^−1^, Tyr^−2^, and SemiQ. At an alkaline pH, SemiQ is singly deprotonated; for this reason, we have performed calculations for SemiQ^−1^.

We have started the analysis of silver–Tyr interactions by placing Ag atoms near the active sites of Tyr^−1^. Four sites of Ag attraction have been established: carboxylate (COO^−^), hydroxyl, phenyl, and amino group. We have found that the most stable complex is between Ag and COO^−^.

Additionally, we have found the atoms responsible for Tyr^−1^ binding with silver ions. We have established five sites of Ag^+^ attraction: carboxylate, hydroxyl, phenol ring, amino group (all four are monodentate), carboxylate, and phenol simultaneously (bidentate).

**Figure 3:**
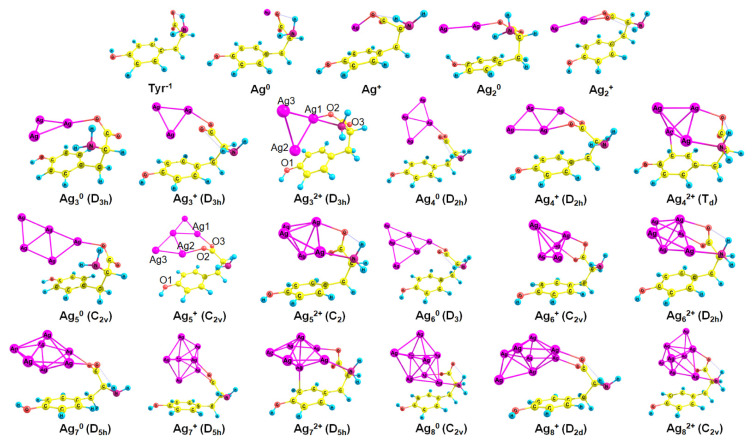
The established complexes of Ag_n_^q^ (*n* = 1–8, q = 0–2) NCs and Tyr^−1^ (PBE-D3 optimization); the point group of a cluster is written in brackets.

The most stable complex for each Agnq–Tyr-1 system (*n* = 1–8, q = 0–2) is demonstrated in Figure 3. Neutral NCs primarily interact with the carboxylate. However, the second site of attraction for Ag20 is the hydroxyl (the premiere Ag–O bond length is 2.17 Å, Ag–H distance is 2.56 Å), and for Ag80 is the amino group (the Ag–O bond length is 2.46 Å, Ag–N distance is 2.34 Å). Additionally, π-interactions are observed for some complexes (for example, in the Ag40–Tyr-1 complex, the distance between Ag and one of the carbon atoms of the phenol is measured to be 2.42 Å). The predominance of Ag–O over Ag–N bonding is not typical for silver cluster complexes of guanine [58] and pterin [60], but is normal for amino acid complexes with silver [33,38].

For cationic clusters, the COO^−^ group is also the most favorable for the interaction with Ag NCs (Figure 3). In addition, Ag_n_^+^ clusters reveal π-binding with the phenol ring: to a greater (Ag_7_^+^: length of Ag–C distance equals 2.42 Å) or lesser extent (Ag_2_^+^: d(Ag–C) = 2.84 Å). The NH_2_ and OH groups are inactive in Ag_n_^+^ binding with Tyr^−1^, whereas in the case of the pterin–Ag_n_^+^ complexes, Ag–N bonding predominates [60].

Tyr^−1^ binds to dicationic NCs like a tridentate chelating agent; it forms bonds with carboxylate, amino group, and phenol. Interactions of Ag with Tyr become more intense since more functional groups are involved in the growth of the cluster charge (Figure 3).

In most cases (Ag_4_^q^, Ag_6_^q^, Ag_7_^q^), the cluster geometry does not change upon the interaction with Tyr^−1^. However, in some cases (Ag_3_^2+^, Ag_5_^+^, Ag_8_^0^, and Ag_8_^2+^), it changes significantly (see Figure 2 and Figure 3 for comparison). With the attachment of Tyr^−1^, Ag_3_^2+^ alters the geometry from linear (D_∞h_) to triangular (D_3h_). Ag_5_^+^ changes its geometry from D_2d_ to C_2v_ upon the interaction with Tyr^−1^. Ag_8_^0^ changes the symmetry point group from T_d_ to C_2v_, and Ag_8_^2+^ alters geometry from D_2d_ to C_2v_.

The situation with the geometry of the Tyr^−2^ complexes is similar to that of the Tyr^−1^ systems except that Ag NCs form an additional Ag–O^–^ bond with the Tyr^−2^ phenol. All clusters have the same symmetry point group, as in the case of the Ag_n_^q^–Tyr^−1^ complexes (Figure S1).

Regarding SemiQ^−1^ and silver clusters, isolated Ag_8_^0^ changes the symmetry from T_d_ to C_s_ upon the addition of SemiQ^−1^, whereas Ag_8_^2+^ changes the geometry from D_2d_ to C_2v_ (Figure S2). As in other cases (Tyr^−1^ and Tyr^−2^), Ag_3_^2+^ changes the geometry from linear to triangular upon the interaction with the ligand. It should be mentioned that SemiQ^−1^ forms Ag–O bonds using the quinone similar to Tyr^−2^ and phenol.

Therefore, the change of cluster geometry during the interplay with Tyr can be used for the experimental detection of Tyr, but generally, the structure of silver NCs does not change significantly with attachment of Tyr^−1^, Tyr^−2^, or SemiQ^−1^.

### 3.3. Binding Energies

The PBE-D3/6-31G(d,p),LANLTZ method has shown sufficient precision in the estimation of Ag-amino acid binding energy [38]. The PBE-D3/6-31G(d,p) method is also efficiently used for guanine–Ag interactions [61]. For this reason, we have used this approach in our investigation.

**Figure 4:**
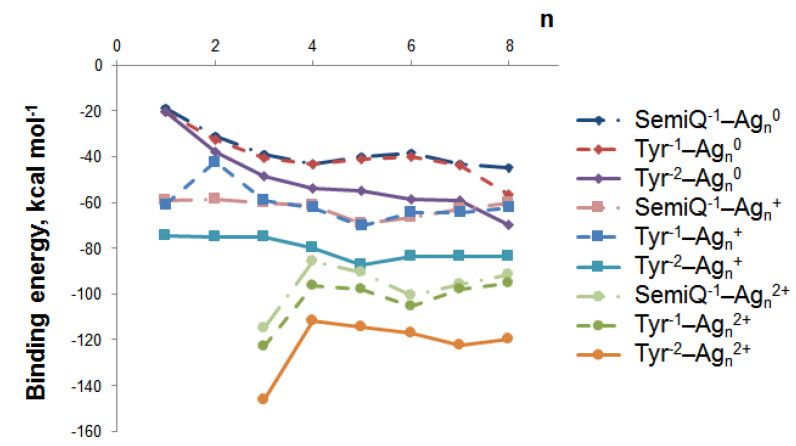
The binding energy between tyrosine and silver nanoclusters Ag_n_^q^ depends on the cluster size (*n*) and charge (q).

We have established the geometry of 66 tyrosine–silver complexes (3 forms of Tyr and 22 nanoclusters). Obviously, the binding energy between Ag NCs and Tyr increases upon H^+^ detachment (Figure 4); with the increment of Tyr charge from −1 to −2, electrostatic interactions between Ag NCs and Tyr increase. The highest interaction energy is observed for the tyrosine complexes of Ag_3_^2+^ NC; the binding energy is equal to −115.1, −122.8, and −146.5 kcal mol^−1^ for SemiQ^−1^, Tyr^−1^, and Tyr^−2^, respectively.

Cationic and dicationic silver clusters form much more stable complexes with Tyr^−1^ than the neutral NCs (Figure 4), and for this reason, they should be regarded more precisely. The Ag_5_^+^ cluster is of particular interest due to its highest interaction energy with tyrosine among the clusters with charges of 0 and +1. For SemiQ^−1^, Tyr^−1^, and Tyr^−2^, the energy of interaction with Ag_5_^+^ equals −69.5, −70.2, and −87.3 kcal mol^−1^, respectively. The binding energies of SemiQ^−1^ and Tyr^−1^ are more or less similar for Ag_n_^0^ and Ag_n_^+^ clusters (Figure 4).

The increase in the binding energy for the Ag_n_^0^ systems continues up to the number of atoms equal to 8. Obviously, the most stable Ag_n_^0^ NC complex with Tyr^−1^ possesses *n*(Ag) > 8. The attachment of Ag_8_^0^ to Tyr^−1^ (Ag_8_^0^ changes symmetry from Td to C_2v_) is characterized by an interaction energy equal to –56.5 kcal mol^−1^. The biding energies of Ag_8_^0^ with Tyr^−2^ and SemiQ^−1^ are also the highest among neutral NCs: −69.7 and −44.9 kcal mol^−1^, respectively.

Therefore, the Ag_3_^2+^ cluster possesses the highest binding energy with all three ligands. The Ag_5_^+^ NC systems are the most stable among clusters, with a charge equal to 0 or +1. The Ag_3_^2+^ and Ag_5_^+^ clusters are the most prospective for experimental synthesis from a theoretical viewpoint and should be regarded as perspective tools for Tyr biosensor development.

### 3.4. QTAIM Analysis

We have used Bader’s quantum theory “Atoms in Molecules” (QTAIM) [46] to study the nature of Ag–X bonding. The Ag_3_^2+^–Tyr and Ag_5_^+^–Tyr complexes as the most stable systems have been regarded in terms of electron density and its derivatives. We have used several QTAIM parameters that are presented in Table S1:

- The density of all electrons ρ;
- The Laplacian of the electron density ∇^2^ρ;
- The Lagrangian kinetic energy G;
- The potential energy density V;
- The energy density H.

All BCPs in Table S1 have positive ∇^2^ρ values, which means that all Ag–X interactions are electrostatic. In each case, electrostatic interactions are stabilizing since all the systems have negative H values. Positive ∇^2^ρ and negative H mean that the Ag–X bond is partially covalent and partially electrostatic. Therefore, QTAIM analysis has shown that all bonds between tyrosine (Tyr^−1^, Tyr^−2^, and SeminQ^−1^) and silver NCs (Ag_3_^2+^ and Ag_5_^+^) are partially covalent and partially electrostatic.

In the Ag_3_^2+^–Tyr^−1^ system, the bond between Ag and N (23.9 kcal mol^−1^) is slightly stronger than the Ag–O bond (22.4 kcal mol^−1^). Presumably, the charge –1 of the ligand is more or less uniformly distributed between Tyr^−1^ atoms. The bonding between N and Ag is stronger than that between O and Ag, which is well known for amino acids [33,38] and nucleobases [39]. Regarding the Ag_5_^+^–Tyr^−1^ complex, the Ag–O3 bond energy is higher than the Ag–O2 energy (for atom numbering, see Figure 3): 24.8 kcal mol^−1^ and 19 kcal mol^−1^, respectively: apparently, O2 has to compete for vacant Ag orbitals with the phenol ring.

H is equal to −0.0028 for both the Ag bond with the phenol oxygen (Ag–O1) and for one of the carboxylate oxygens (Ag–O2) in the Tyr^−2^–Ag_3_^2+^ complex. However, the absolute value of V (−0.0621 and −0.0684, respectively), as well as the bond energy (19.5 kcal mol^−1^ and 21.5 kcal mol^−1^, respectively), are higher for O2. This is in agreement with our previous observations that silver tends to form the bond first with carboxylate and only then with phenolate [33,38]. The Tyr^−2^–Ag_5_^+^ complex demonstrates that the interactions between Ag and carboxylate (E_bond_(Ag–O2) = 23.2 kcal mol^−1^, E_bond_(Ag–O3) = 21.6 kcal mol^−1^) are slightly stronger than those between Ag and phenolate (E_bond_(Ag–O1) = 20.8 kcal mol^−1^).

Regarding the SemiQ^−1^–Ag_3_^2+^ system, NH_2_ has a priority in Ag binding; the bond energy increases in a row O1 < O2 < N (21 kcal mol^−1^, 22 kcal mol^−1^ kcal mol^−1^, and 24 kcal mol^−1^, respectively), demonstrating that silver attaches primarily to the NH_2_ group, then to carboxylate, and only than to semiquinone. For the SemiQ^−1^–Ag_5_^+^ complex, the Ag–O2 (26.3 kcal mol^−1^) and Ag–O3 (26.8 kcal mol^−1^) bond energies are 2.3 times higher than the E_bond_ of Ag–O1 (11.4 kcal mol^−1^).

### 3.5. Absorption Spectra

We have calculated the absorption spectra for the most energetically favorable complexes of silver NCs and Tyr. Geometry optimization has been performed with the MP2/6-31G(d,p),LANLTZ method, which has previously demonstrated high effectiveness on ligand-protected Ag NCs [48]. The first 10 transitions in the electronic absorption spectra have been calculated with TDDFT, in particular the M062X/def2-TZVP method, which has proved its efficacy in simulating the spectra of similar silver–organic systems [51].

With the attachment of Tyr^−1^, Ag_3_^2+^ changes its symmetry point group from linear (D_∞h_) to triangular (D_3h_). Ag_5_^+^ also changes its symmetry: from D_2d_, or X-like in the isolated state (Figure 2) to a C_2v_ or W-like structure (Figure 3) upon the interaction with Tyr^−1^. Obviously, this change in cluster symmetry leads to a significant modulation of the cluster absorption spectra.

**Figure 5:**
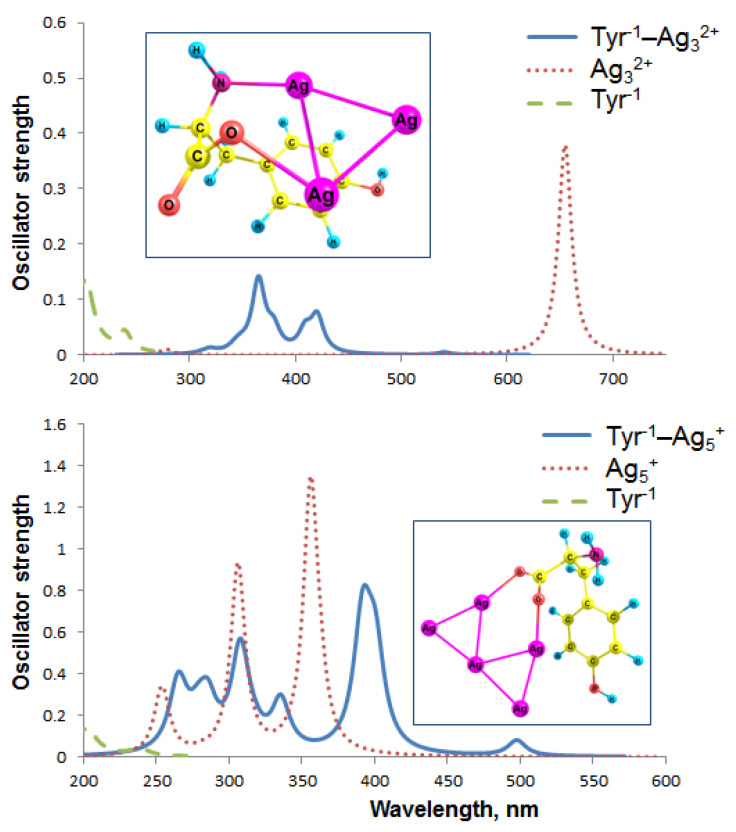
Absorption spectra of the Tyr^−1^ complexes with Ag nanoclusters according to the TDDFT M062X/def2-TZVP method. Lorentzian broadening with a bandwidth at ½ of the height (FWHM) equal to 15 nm has been used. Geometry of the complexes is presented in the insets.

Our calculations accurately reproduce the experimental absorption spectrum of deprotonated Tyr^−1^. The predominant band in the spectral region of 230–300 nm is located at 239 nm and 241 nm [62] according to our calculations and experiment, respectively. Isolated linear Ag_3_^2+^ has a theoretical UV-Vis spectrum with a single band located at 655 nm (the oscillator strength f_OSC_ equals 0.379) (Figure 5). With the addition of Ag_3_^2+^, the spectrum of Tyr^−1^ changes dramatically; a long-wave maximum is located at 541 nm (0.005), whereas the major peak is at 365 nm (0.130). Apparently, the interaction of the Ag_3_^2+^ cluster with Tyr^−1^ is prospective for the colorimetric detection of tyrosine.

According to Kasha’s rule, fluorescence occurs from the S_1_ state. Since the f_OSC_ of the S_0_ → S_1_ transition is low (0.005), S_1_ → S_0_ fluorescence should also occur with low intensity. Therefore, fluorimetric detection of Tyr^−1^ using Ag_3_^2+^ NC is hardly possible.

The major maximum of the Tyr^−1^–Ag^5+^ complex is at 393 nm (f_OSC_ = 0.828), and the long-wave maximum is at 498 nm (0.075). The spectrum of isolated Ag_5_^+^ has a long-wave maximum at 519 nm with zero oscillator strength, whereas the predominant transition is characterized by λ_max_ = 356 nm and f_OSC_ = 1.255. The Tyr^−1^–Ag^5+^ complex as a whole is strongly red-shifted in comparison to Tyr^−1^ (Figure 5). Apparently, Ag_5_^+^ can be used, if not for the luminescent, then for the colorimetric detection of Tyr.

The frontier molecular orbitals (MOs) participating in S_0_ → S_1_ transitions are presented in Figure S3. The highest occupied (HOMO) and singly occupied (SOMO) orbitals of the Tyr^−1^–Ag_3_^2+^ system relate to the ligand–metal transition. The HOMO and the lowest unoccupied (LUMO) orbitals of the Tyr^−1^–Ag_5_^+^ complex relate to the metal–metal transition.

**Figure 6:**
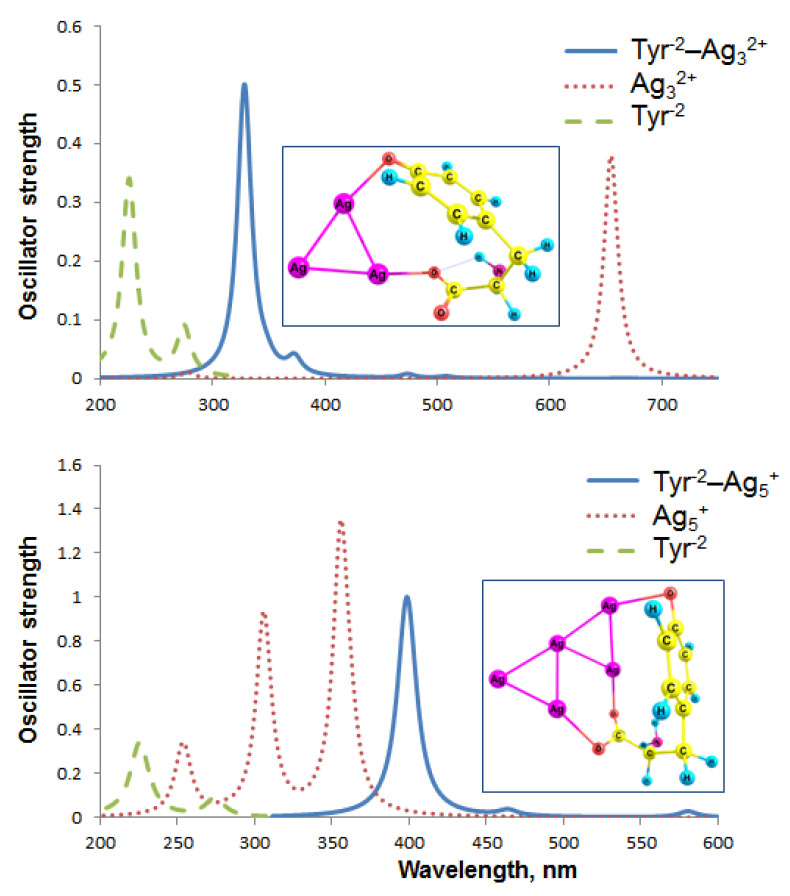
Absorption spectra of the Tyr^−2^ complexes with Ag nanoclusters.

The Tyr^−2^–Ag_3_^2+^ spectrum is dominated by a strong absorption band at 328 nm with a f_OSC_ equal to 0.5 (Figure 6). The S_0_ → S_1_ transition is located in the infrared region (1180 nm) and has zero oscillator strength; therefore, we suppose this complex does not fluoresce. The predominant band in the UV-vis spectrum of pure Tyr^−2^ is located at 226 nm (0.337). Thus, the Tyr^−2^ spectrum significantly transforms due to the silver–ligand interactions; Ag_3_^2+^ forms bonds not only with the carboxylate, but also with the phenolate, which should provide selectivity to identify Tyr in the amino acid mixture. For this reason, a combination of pH > 10.5 and Ag_3_^2+^ cluster seems to be highly promising for the colorimetric detection of Tyr.

Interestingly, transitions with zero f_OSC_ are predominant among the first 10 transitions of the Tyr^−2^–Ag_5_^+^ absorption spectrum. For this reason, multiple bands present in the Tyr^−1^–Ag_5_^+^ spectrum (Figure 5) are absent from the Tyr^−2^–Ag_5_^+^ spectrum (Figure 6). Thus, the oscillator strength of the first transition (853 nm) in the Tyr^−2^–Ag_5_^+^ spectrum equals zero, which makes this system improper for fluorescent Tyr detection. Apparently, the difference between the Tyr^−1^–Ag_5_^+^ and Tyr^−2^–Ag_5_^+^ spectra is determined by additional interactions of Ag with the phenolate, which does not occur in Tyr^−1^–Ag_5_^+^. The predominant transition in the Tyr^−2^–Ag_5_^+^ UV-vis spectrum is located at 399 nm (f_OSC_ = 1), which is similar to the major band of Tyr^−1^–Ag_5_^+^ at 393 nm (0.828).

The bare SemiQ^−1^ has a major band at 229 nm (f_OSC_ = 0.307). The SemiQ^−1^–Ag_3_^2+^ spectrum is dominated by a band at 360 nm (0.11), whereas the S_0_ → S_1_ transition is located at 580 nm and has an oscillator strength equal to 0.001 (Figure S4). Therefore, the complex should not fluoresce. The 360-nm band possesses too low an intensity (f_OSC_ = 0.11), even compared to the isolated SemiQ^−1^ (0.307). For this reason, the SemiQ^−1^–Ag_3_^2+^ system is not appropriate for colorimetric Tyr detection either.

The first, second, third, fifth, eighth, and tenth transitions in the SemiQ^−1^–Ag_5_^+^ spectrum have zero oscillator strength, which makes this system unacceptable for luminescent Tyr detection. The predominant transition is located at 394 nm (f_OSC_ = 1.007), which is similar to the major band of Tyr^−1^–Ag_5_^+^ (λ_max_ = 393 nm, f_OSC_ = 0.828) and Tyr^−2^–Ag^5+^ (λ_max_ = 399 nm, f_OSC_ = 1).

MOs participating in the main transitions of the absorption spectra of the Tyr^−2^ and SemiQ^−1^ complexes are presented in Figure S5. The HOMO and LUMO orbitals of the Tyr^−2^–Ag_3_^2+^ system relate to the ligand–metal π → π* transition responsible for the major band in the UV-vis spectrum (328 nm, f_OSC_ = 0.5, S_0_ → S_10_). Regarding the SemiQ^−1^–Ag_3_^2+^ complex, the HOMO and SOMO orbitals are responsible for the S_0_ → S_1_ transition (ligand–metal charge transfer).

Thus, neither of the described six complexes is prospective for the colorimetric/luminescent Tyr detection, except the Tyr^−2^–Ag_3_^2+^ system, which has the potential to be used for colorimetric Tyr detection at pH > 10.5.

### 3.6. Vibrational Spectroscopy

Vibrational spectroscopy of ligand-protected metal nanoclusters offers opportunities for researchers in different fields, including biosensors [63]. We have evaluated the possibility of Tyr detection using vibrational and surface-enhanced Raman spectroscopy (SERS). In order to estimate the potential benefits of these methods on Tyr–Ag_n_^q^ systems, the infrared (IR) and Raman spectra of the complexes have been calculated with the B3LYP-D3/6-31G(d,p),LANLTZ method. The B3LYP, together with dispersion correction and the 6-31G(d,p) basis set, gives correct spectra as compared to other DFT functionals and even the MP2 method [52].

**Figure 7:**
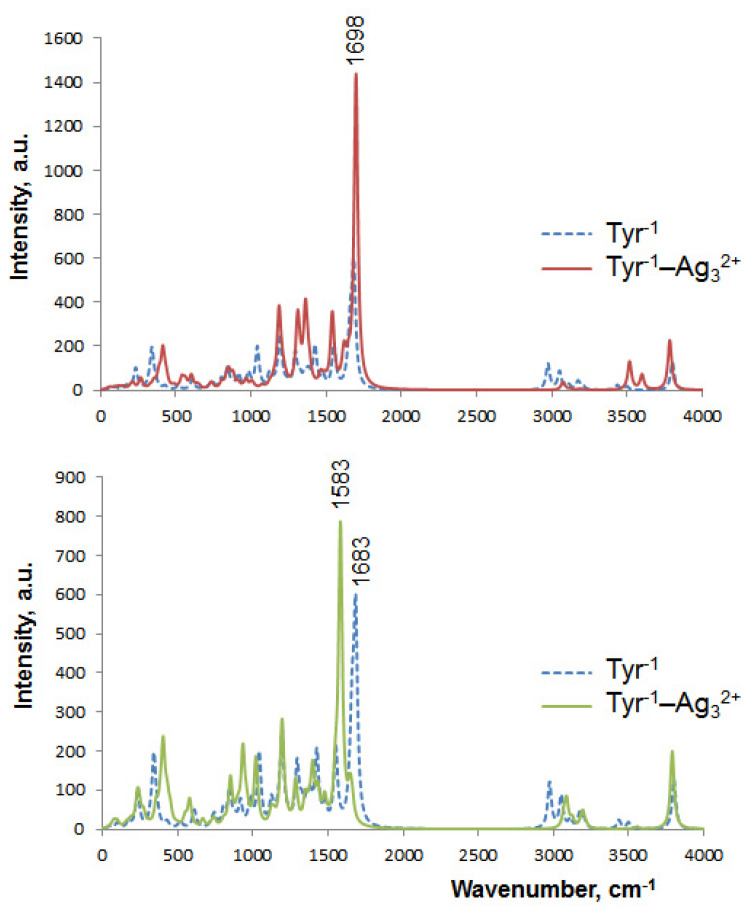
Infrared spectra of bare Tyr^−1^ and its complexes with silver according to the B3LYP-D3 method. Lorentzian broadening, with a bandwidth at ½ of the height equal to 30 cm^−1^, has been used.

IR spectra of the isolated clusters possess too low intensity and low frequency transitions; therefore, we do not regard them. The isolated Tyr^−1^ can be characterized by a major band at 1683 cm^−1^ (606 a.u.), corresponding to asymmetric stretching of the carboxylate (Figure 7). The stretching of the amino group is characterized by a vibration frequency equal to 1658 cm^−1^. The twisting of the β-CH_2_ group has a frequency of 1174 cm^−1^.

Quite significant IR spectrum transformations occur when silver NCs are added to Tyr^−1^. Upon the addition of Ag_3_^2+^ (in particular, through the Ag–O2 bonding), the main transition (the COO asymmetric stretching) blue-shifts from 1683 cm^−1^ to 1698 cm^−1^; its intensity increases by a factor of 2.4 (from 606 to 1438 a.u.). Other differences in the Tyr^−1^–Ag_3_^2+^ spectrum are minor compared to Tyr^−1^ (Figure 7).

When the Ag_5_^+^ cluster is added to bare Tyr^−1^, the frequency of the 1683 cm^−1^ transition decreases to 1583 cm^−1^, while the intensity increases by 30% (from 606 to 785 a.u.). It is worth noting that both carboxylate oxygens attach to Ag in the Tyr^−1^–Ag_5_^+^ complex. Otherwise, the changes in the IR spectrum are insignificant as compared to the isolated Tyr^−1^.

The major band of the Tyr^−2^ IR spectrum has a frequency of 1568 cm^−1^ (680 a.u.) and corresponds to the phenolate C–O bond stretching. Another intense transition is located at 1692 cm^−1^ (589 a.u.) and associated with the asymmetric stretching of the COO- group. The Tyr^−2^ IR spectrum does not change significantly upon the addition of Ag NCs (Figure S6). With the addition of Ag_3_^2+^, the major bands are red-shifted to 1525 cm^−1^ and 1610 cm^−1^, respectively, and possess diminished intensities. On the contrary, the attachment of Ag_5_^+^ increases the band at 1568 cm^−1^ by 14%, 1579 cm^−1^ (778 a.u.). Ag_5_^+^ interacts with all three oxygen atoms of Tyr^−2^.

Quite intense transitions are located in the region of 3000–3500 cm^−1^ of the Tyr^−2^ spectrum. The main bands at 3007 cm^−1^ (185 a.u.) and 3148 cm^−1^ (214 a.u.) relate to the α-C–H and phenol C–H bond stretching, respectively. However, upon the attachment of Ag_3_^2+^, these bands shift to 3094 cm^−1^ (48 a.u.) and 3199 cm^−1^ (42 a.u.); thus, intensities substantially diminish 4- and 5-fold, respectively. The same is true for these bands and the Tyr^−2^–Ag_5_^+^ complex; upon the cluster addition, the bands at 3007 cm^−1^ and 3148 cm^−1^ of the bare Tyr^−2^ blue-shift to 3070 cm^−1^ (113 a.u.) and 3170 cm^−1^ (120 a.u.) with 39% and 44% intensity reduction, respectively. Ag does not interact with C–H groups either in the case of Ag_3_^2+^ or in the case of Ag_5_^+^.

Significant chemical enhancement is observed in the region of 800–1000 cm^−1^. The IR spectrum of bare Tyr^−2^ is characterized by 845 cm^−1^ (133 a.u.) and 955 cm^−1^ (266 a.u.) bands. With the attachment of Ag_3_^2+^ to carboxylate and O of the phenol, these bands are grouped into a single band with a maximum at 908 cm^−1^ (334 a.u.), which gives a 26% chemical enhancement (Figure S6); the main transition involved in this band corresponds to the amino group wagging. The addition of Ag_5_^+^ does not bring significant changes in this region of the spectrum.

Probably the most dramatic transformations occur in the region of 100–200 cm^−1^. With the addition of Ag_3_^2+^, a peak arises at 188 cm^−1^ (180 a.u.); whereas with the attachment of Ag_5_^+^, a band appears at 217 cm^−1^ (205 a.u.). In both cases, this corresponds to the NH_2_ group twisting. Apparently, these transitions are not intense enough for the experimental determination of Tyr^−2^.

Therefore, a combination of vibrational spectroscopy, silver nanoclusters, and pH > 10.5 should not be considered as a prospective methodology for Tyr detection.

**Figure 8:**
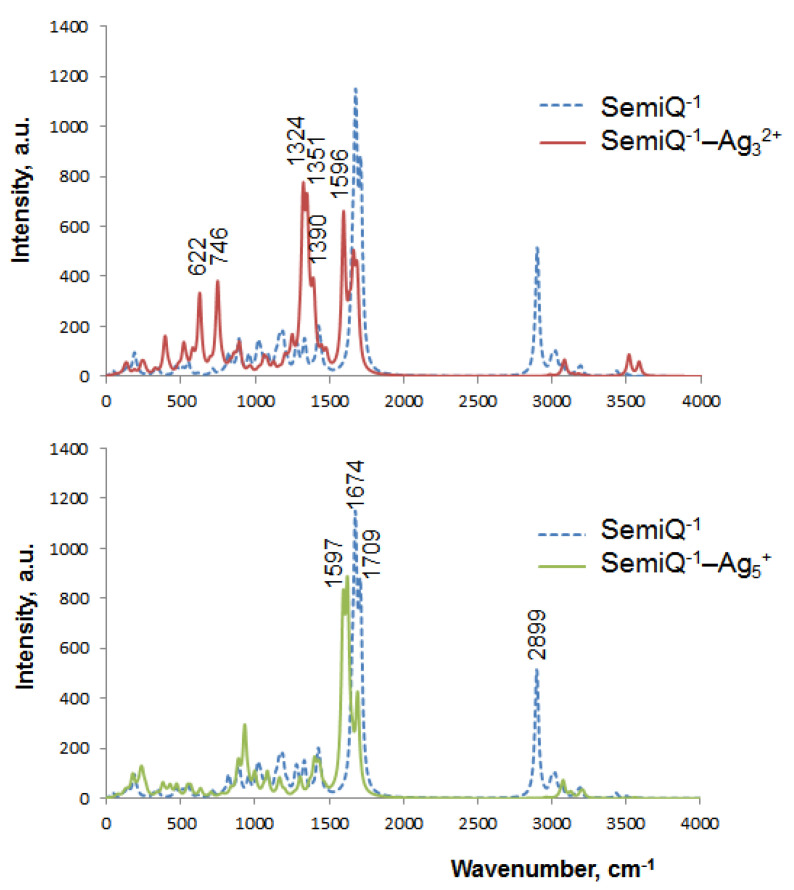
Infrared spectra of the isolated SemiQ^−1^ and its complexes, SemiQ^−1^–Ag_3_^2+^ and SemiQ^−1^–Ag_5_^+^.

The vibrational spectrum of SemiQ^−1^ (Figure 8) is characterized by a major band with two maxima: 1674 cm^−1^ (1149 a.u.) and 1709 cm^−1^ (875 a.u.). The first maximum corresponds to the asymmetric stretching of the carboxylate, whereas the second one relates to the phenol C=O stretching. These two maxima significantly diminish upon the addition of silver NCs. With the attachment of Ag_3_^2+^, these peaks shift to 1596 cm^−1^ (659 a.u.) and 1665 cm^−1^ (501 a.u.), respectively; thus, the intensity decreases by 43% in both cases. Upon the interaction with Ag_5_+, these peaks transform into three maxima: 1597 (830 a.u.), 1623 cm^−1^ (889 a.u.), and 1691 cm^−1^ (425 a.u.); the intensities of the two main maxima decrease by 23% and 51%, respectively, upon the addition of Ag_5_^+^. The first maximum (the asymmetric stretching of COO^–^) is directly influenced by Ag: Ag_5_^+^ forms bonds with both carboxylate oxygens, whereas the C=O group of the phenol does not interact with Ag_5_^+^.

Another intense transition in the SemiQ^−1^ spectrum is located at 2899 cm^−1^ (514 a.u.) and corresponds to the γ-C–H stretching. This band disappears completely upon the addition of silver clusters (Figure 8).

However, the most dramatic changes occur in the spectral region of 1200–1500 cm^−1^ with the addition of Ag_3_^2+^ to bare SemiQ^−1^. The isolated SemiQ^−1^ spectrum possesses three low-intensity bands in this area, whereas SemiQ^−1^–Ag_3_^2+^ possesses an intense band with three maxima (Figure 8): 1324 cm^−1^ (775 a.u.), 1351 cm^−1^ (733 a.u.), and 1390 cm^−1^ (394 a.u.). The band dominates in the IR spectrum of the SemiQ^−1^–Ag_3_^2+^ complex. The peaks relate to the H–α-C–C scissoring (not Ag-associated), the NH_2_ twisting (N is bound to Ag), and the α-C–H rocking (not Ag-associated), respectively. Apparently, the band at 1324 cm^−1^ can be exploited for selective and efficient vibrational spectroscopy (as well as Raman and SERS) detection of SemiQ^−1^ since 2.5-fold intensity enhancement occurs as compared to the band at 1361 cm^−1^ (311 a.u.) of the bare semiquinone. Moreover, Ag_3_^2+^ interacts with all three functional groups of SemiQ^−1^: phenolate, amino group, and carboxylate (Figure S2), which makes it a highly specific and selective SemiQ biosensor. The SemiQ^−1^–Ag_5_^+^ and SemiQ^−1^ spectra do not differ significantly in the region of 1200–1500 cm^−1^ (Figure 8).

With the addition of Ag_3_^2+^, two bands appear: 622 cm^−1^ (334 a.u.) and 746 cm^−1^ (381 a.u.). Either the SemiQ^−1^–Ag_5_^+^ or bare SemiQ^−1^ spectrum does not possess these peaks. Presumably, these bands are not pronounced enough for efficient use in vibrational spectroscopy for the detection of Tyr.

The IR spectrum of the Tyr^−1^–Ag_5_^+^ system is the most similar to the experimental one of tyrosine in the presence of silver nanoparticles at pH 10 [64]. The main transition in our simulated Tyr^−1^–Ag_5_^+^ spectrum is located at 1583 cm^−1^ (responsible for the asymmetric COO- stretching), whereas the experimental one is at 1596 cm^−1^. The twisting of the amino group has a frequency of 1177 cm^–1^, and the experimental one is 1140 cm^−1^ [64]. Thus, our simulated IR spectra are in good agreement with the experimental ones. The use of vibrational and Raman spectroscopy, along with silver NCs, seems to be promising for the detection of tyrosine.

It seems that the most significant signal enhancement occurs upon the attachment of Ag_3_^2+^ to Tyr^−1^ carboxylate (the band at 1698 cm^−1^ relates to the O–C–O asymmetric stretching, see Figure S7). However, this transition at 1698 cm^−1^ is not selective enough; carboxylates are present in multiple compounds. Apparently, a combination of Raman spectroscopy and the Ag_3_^2+^ NC synthesis on Tyr should be considered a prospective and selective method of Tyr detection; the emergence of the band at 1324 cm^−1^ (Figure 8) upon the interaction of the cluster with SemiQ^−1^ looks prospective for experimental amino acid detection.

**Figure 9:**
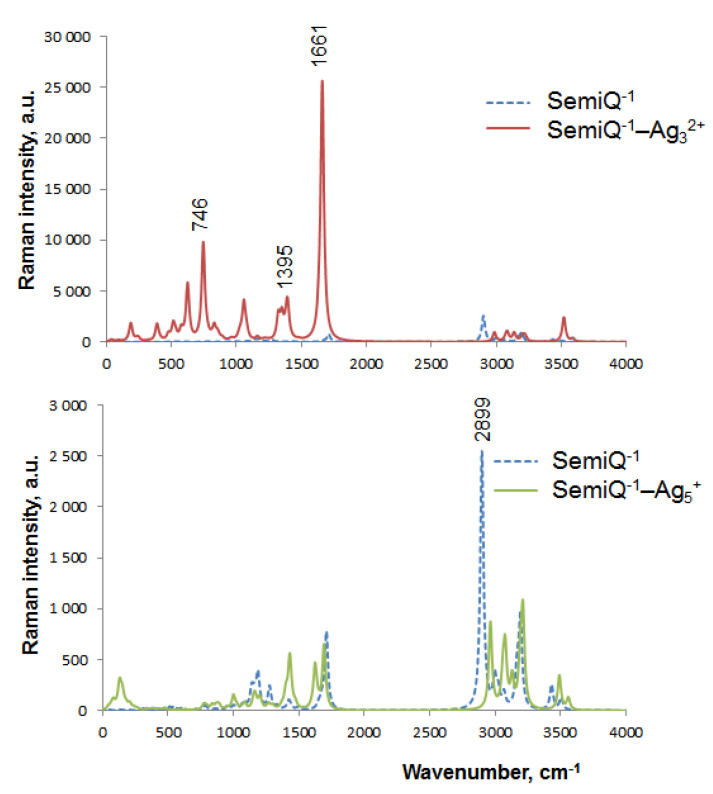
Raman spectra of bare SemiQ^−1^ and its complexes (SemiQ^−1^–Ag_3_^2+^ and SemiQ^−1^–Ag_5_^+^) according to the B3LYP-D3 method (Lorentzian broadening with a bandwidth at ½ of the height equals 30 cm^−1^).

Additionally, we have calculated Raman spectra of SemiQ^−1^ attached to silver NCs as the most promising tools for Tyr detection by vibrational spectroscopy. Upon the interaction of SemiQ^−1^ with Ag_3_^2+^, a strong chemical enhancement with the emergence of the bands at 746 cm^−1^ (9558 a.u.) and 1661 cm^−1^ (25228 a.u.) is observed in the Raman spectrum (Figure 9). The first maximum (scissoring of COO^–^) is directly influenced by silver; Ag_3_^2+^ forms a bond with one of the carboxylate oxygens. The C–O bond stretching of the second carboxylate oxygen is responsible for the major peak at 1661 cm^−1^. The most intriguing band of the SemiQ^−1^–Ag_3_^2+^ vibrational spectrum has a low intensity in the Raman spectrum; its major peak at 1389 cm^−1^ possesses 3966 a.u. The Raman spectrum of the pure SemiQ^−1^ is characterized by a major band with a maximum at 2899 cm^−1^ (2547 a.u.), which corresponds to the γ-C–H bond stretching. With the attachment of Ag_5_^+^, this peak shifts to 2984 cm^−1^ and diminishes (920 a.u.); thus, its intensity decreases by 64%. Therefore, Ag_3_^2+^ NC seems to be an efficient tool for Raman and SERS Tyr semiquinone detection.

### 3.7. Natural Bond Orbital Analysis

According to the chemical theory of SERS, the chemical enhancement exploited by Raman spectroscopy is largely determined by charge transfer (CT). Three types of charge transfer can be responsible for the SERS chemical enhancement: ground state charge transfer (GSCT), photoinduced CT (PICT), and intermolecular CT [65]. Thereby, we have performed a natural bond orbital (NBO) analysis to evaluate the possibility of GSCT.

Since we are interested in NBO analysis in relation to Raman chemical enhancement, we have performed calculations using the B3LYP-D3 method, as in the case of the IR and Raman spectra simulation. The B3LYP/6-31(d,p) method has already proved its efficacy in the NBO analysis [66,67].

The natural charges of Ag atoms and tyrosine atoms X are presented in Table S2 (q_Ag_ and q_X_, respectively). Silver atoms have a positive charge, whereas N and O have a negative charge. This means that Ag–X bonds have an electrostatic nature, which has already been demonstrated with the QTAIM analysis. The Ag_3_^2+^ cluster charge (q_cluster_) lies in the range of 0.7–1.2, which means it oxidizes all forms of tyrosine.

The Ag–X bond orders were estimated using the Wiberg bond index. The Ag1–O2 (see Figure 3 for atom numbering) bond index is the highest for the Tyr^−1^–Ag_3_^2+^ complex (0.611). In the SemiQ^−1^–Ag_3_^2+^ complex, the Ag1–O2 bond index is smaller (0.58), as well as in the Tyr^−2^–Ag_3_^2+^ system (0.474). The Ag1–N Wiberg index value is similar for the Tyr^−1^–Ag_3_^2+^ complex (0.41) and the SemiQ^−1^–Ag_3_^2+^ system (0.458). The Ag2–O1 bond index is similar for SemiQ^−1^–Ag_3_^2+^ (0.504) and Tyr^−2^–Ag_3_^2+^ (0.565). Therefore, from the point of view of the Wiberg bond index, the silver interactions with carboxylate dominate in the Tyr^−1^ and SemiQ^−1^ complexes of Ag_3_^2+^, whereas the Ag binding to the O1 atom of the phenolate prevails in the Tyr^−2^–Ag_3_^2+^ system.

The results of the natural population analysis (NPA) are demonstrated in Table S2. In a complex with Tyr, all atoms of Ag_3_^2+^ are positive, whereas Ag_5_^+^ atoms have nearly zero charges (q_Agn_). Thus, the silver atom charges decrease in the complex compared to bare clusters. The Ag_3_^2+^ and Ag_5_^+^ cluster charge (q_cluster_) lies in the range of +0.7–+1.1 and −0.4–+0.1 when the cluster is in the complex with Tyr, which means that the clusters oxidize all forms of tyrosine, attracting its electrons. Apparently, the Ag–O pairs of the Tyr–Ag_3_^2+^ system undergo electrostatic attraction, whereas the Ag–O bonds of Tyr–Ag_5_^+^ possess a slight electrostatic repulsion, as the NPA data show; both Ag and O possess a negative charge (Table S2).

The NBO analysis provides valuable information when studying intramolecular bonding and interaction between bonds. The main electron donor orbital and acceptor orbital occupancies and the interaction stabilization energy (E^(2)^) resulting from the B3LYP-D3 NBO analysis are presented in Table 1. The larger the value of E^(2)^, the more intense the interaction between the electron donor orbital *i* and the electron acceptor orbital *j*, which means a greater donation tendency from electron donor to electron acceptor [68]. Delocalization of the electron density between an occupied bond or a lone pair of NBO orbitals and a formally unoccupied antibond NBO orbital demonstrates a stabilizing donor–acceptor interaction. The intramolecular interaction is formed by the orbital overlap between *n*, n*, σ, σ*, π, and π* orbitals. The intramolecular GSCT causes stabilization of the whole system. These interactions lead to an increase in electron density in the antibonding orbital that loosens the respective bonds.

Table 1 shows E^(2)^ and q_CT_ for the main ground state charge transfer with the involvement of silver. Most interactions occur between the *n* and σ orbitals, whereas the participation of the π orbitals in CT is rare. The σ_O2-Ag1_ → n*_O3-C_ charge transfer (Figure S8) occurs in the Tyr^−1^–Ag_3_^2+^ complex (E^(2)^ = 5.77 kcal mol^−1^ and q_CT_ = 0.02). This GSCT is responsible for the band at 1698 cm^−1^ in the IR spectrum of the Tyr^−1^–Ag_3_^2+^ system (Figure 7).

One of the main metal–ligand CTs of the SemiQ^−1^–Ag_3_^2+^ complex is a σ_O2-Ag1_ → n*_C-O3_ delocalization (Figure S8), which is characterized by E^(2)^ = 6.12 kcal mol^−1^ and q_CT_ = 0.022. Obviously, this CT is responsible for the peak at 1661 cm^−1^ in the Raman spectrum (Figure 9). The σ_O1-Ag2_ → σ*_C(O1)-C_ CT is also observed in the SemiQ^−1^–Ag_3_^2+^ complex: E^(2)^ = 5.08 kcal mol^−1^ and q_CT_ = 0.02. This GSCT relates to the C–O1 stretching and is characterized by a transition at 1595 cm^−1^. The n_N_ → n*_Ag1_ CT (E^(2)^ = 32.35 kcal mol^−1^, q_CT_ = 0.225) relates to the transition at 1350 cm^−1^ (the NH_2_ group twisting) and the major band of the SemiQ^−1^–Ag_3_^2+^ system vibrational spectrum (Figure 7). This transition at 1350 cm^−1^ and the band at 1395 cm^−1^ as a whole (Figure 9) can be used for the Raman spectroscopy detection of Tyr.

The largest stabilization energy among the SemiQ^−1^–Ag_n_^q^ complexes equals 55.95 kcal mol^−1^ and corresponds to a n_O3_ → n*_Ag1_ CT (Figure S8) of the SemiQ^−1^–Ag_5_^+^ system. Presumably, this GSCT relates to the peak at 1597 cm^−1^ (the carboxylate asymmetric stretching) in the IR spectrum; however, its intensity is extremely low in the Raman spectrum (14 a.u.).

The highest values of stabilization energy E^(2)^ and the amount of transferred charge q_CT_ among all complexes have been established for the σ_O1-Ag3_ → n*_C(O1)_ charge transfer in the Tyr^−2^–Ag_5_^+^ system: 93.34 kcal mol^−1^ and 1.502, respectively. Apparently, this GSCT is responsible for the band at 1324 cm^−1^ (the C–O1 stretching), which is the second most intense band in the IR spectrum of Tyr^−2^–Ag_5_^+^ (Figure S6). Additionally, in the Tyr^−2^–Ag_5_^+^ complex, the charge is transferred from the σ_O2-Ag2_ bond orbital to the π*_C-O3_ antibond (Figure S8): E^(2)^ = 39.66 kcal mol^−1^ and q_CT_ = 0.174. Presumably, this GSCT is responsible for the transition at 1579 cm^−1^ (the asymmetric carboxylate stretching) in the IR spectrum of the Tyr^−2^–Ag_5_^+^ system (Figure S6).

Therefore, we may conclude that NBO analysis gives valuable information on the ground state CT responsible for the chemical enhancement in the Raman spectra of the Tyr–Ag_n_^q^ systems. The large band at 1661 cm^−1^ (25228 a.u.) observed in the Raman spectrum of SemiQ^−1^–Ag_3_^2+^ (Figure 9) corresponds to the carboxylate interaction with Ag. The nearest band of the SemiQ^−1^ in this region is located at 1710 cm^−1^ (762 a.u.). Thus, Ag_3_^2+^ permits a 33-fold enhancement and a signal shift by 49 cm^−1^.

The photoinduced CT may also be responsible for the SERS chemical enhancement [65]. The main transitions in the regarded Tyr–Ag systems are either ligand–metal or metal–metal (Figures S3 and S5). Apparently, the metal–metal transitions are not prospective for SERS. We suppose that a combination of the NBO analysis and the ground state CT evaluation gives more reliable and well interpretable results than the UV-vis spectra simulation and the analysis of molecular orbitals.

## 4. Conclusions

We have performed the quantum–chemical calculations for the silver nanocluster complexes with Tyr^−1^, Tyr^−2^, and SemiQ^−1^. We have shown that the most energetically favorable conformation of tyrosine in a complex differs depending on the cluster size and charge. In all cases, the interactions between silver and organic ligands are partially covalent and partially electrostatic. The complexes of the Ag_3_^2+^ cluster are the most energetically favorable. The Ag_5_^+^ systems are the most stable among NCs, with a charge equal to 0 or +1.

The Ag_3_^2+^ nanocluster seems to be the most promising tool for the colorimetric detection of tyrosine in aqueous solutions with pH > 10.5; the absorption maximum of the Tyr^−2^–Ag_3_^2+^ system is located at 328 nm (f_OSC_ = 0.5), whereas the spectrum of the isolated Tyr^−2^ is completely different. The Tyr^−2^–Ag_3_^2+^ complex appears to be quite specific for the UV-vis detection of Tyr since the cluster interacts with both phenolate and carboxylate of Tyr^−2^. The luminescent detection of tyrosine seems to be less likely due to the low intensity of the lowest energy transition.

The natural bond orbital theory allows one to analyze the main transitions of the SERS spectra in terms of the metal–ligand ground state charge transfer (CT). Thus, the CTs between Ag and the functional groups of SemiQ^−1^ determine the emergence of a band at 1661 cm^−1^ in the spectrum of the SemiQ^−1^–Ag_3_^2+^ complex.

The reduction of Ag^+^ to Ag_3_^2+^ with Tyr^−1^ and the following detection of SemiQ^−1^ with SERS looks prospective. Ag_3_^2+^ interacts with all three functional groups of SemiQ^−1^, phenolate, amino group, and carboxylate (Figure S2), which makes it a highly specific and selective tool for SemiQ detection. The intense band at 1324 cm^−1^ in the vibrational spectrum is a fingerprint of the Tyr–Ag NC complex vibrational spectrum.

For now, theoretical studies of interactions between silver nanoclusters and small organic ligands with involvement of SERS and NBO analysis are few [69]. However, one should not underestimate the importance of such research. Therefore, our results should be beneficial for the experimental determination of aromatic amino acids (Tyr, Phe, DOPA) in biological fluids.

## Figures and Tables

**Table 1:** Second-order perturbation theory analysis of the Fock matrix in the NBO basis for the most stable Tyr–Ag_n_^q^ complexes.

Complex	Donor (i)	Orbital Type	Occupancy	Acceptor (j)	Orbital Type	Occupancy	E^(2)^, kcal mol^−^1	q_CT_
Tyr^−1^–Ag_3_^2+^	C	*n*	0.547	Ag2	n*	0.107	27.31	0.453
Tyr^−1^–Ag_3_^2+^	O2–Ag1	σ	0.956	O3–C	n*	0.024	5.77	0.02
Tyr^−1^–Ag_3_^2+^	Ag2	*n*	0.952	O1–C	π*	0.017	1.92	0.205
SemiQ^−1^–Ag_3_^2+^	O1–Ag2	σ	0.953	C(O1)–C	σ*	0.032	5.08	0.02
SemiQ^−1^–Ag_3_^2+^	O2–Ag1	σ	0.992	C–O3	σ*	0.024	6.12	0.022
SemiQ^−1^–Ag_3_^2+^	N	*n*	0.853	Ag1	n*	0.2	32.35	0.225
Tyr^−2^–Ag_3_^2+^	O2–Ag1	σ	0.838	C–O3	π*	0.05	28.49	0.394
Tyr^−2^–Ag_3_^2+^	O1–Ag2	σ	0.865	C(O1)	n*	0.486	48.32	1.466
Tyr^−1^–Ag_5_^+^	O1	*n*	1.785	Ag3	n*	0.161	14.05	0.059
Tyr^−1^–Ag_5_^+^	O2–Ag2	σ	1.633	C–O3	π*	0.393	40.61	0.174
Tyr^−1^–Ag_5_^+^	O3	*n*	1.653	Ag1	n*	0.392	60.1	0.206
SemiQ^−1^–Ag_5_^+^	C–O1	π	1.909	Ag3	n*	0.212	8.42	0.029
SemiQ^−1^–Ag_5_^+^	O2	*n*	1.696	O2–Ag2	σ *	0.272	50.73	0.177
SemiQ^−1^–Ag_5_^+^	O3	*n*	1.664	Ag1	n*	0.394	55.95	0.19
Tyr^−2^–Ag_5_^+^	O1–Ag3	σ	1.74	C(O1)	n*	0.963	93.34	1.502
Tyr^−2^–Ag_5_^+^	O2–Ag2	σ	1.636	C–O3	π*	0.383	39.66	0.174
Tyr^−2^–Ag_5_^+^	O3	*n*	1.646	Ag1	n*	0.409	51.37	0.188

## Data Availability

Data is contained within the article or supplementary material.

## Supplementary Materials

The following supporting information can be downloaded at: https://www.mdpi.com/article/10.3390/ijms23020634/s1.
