# Supplementary material for: Silver Cluster Interactions with Tyrosine: Towards Amino Acid Detection
Source: Int J Mol Sci. 2022 Jan 6;23(2):634. doi: 10.3390/ijms23020634 (PMC8775517; doi:10.3390/ijms23020634)
Supplement: Supplementary file 1 [file ijms-23-00634-s001.zip › ijms-1541185-supplementary.pdf]

Silver Cluster Interactions with Tyrosine in Aqueous Solutions: Towards Amino Acid Detection

Andrey A. Buglak[a,]*, Alexei I. Kononov[a]

[a] The Faculty of Physics, Saint-Petersburg State University, 199034 St. Petersburg, Russia

* Corresponding author:
andreybuglak@gmail.com (A.A. Buglak)

Supplementary information

Contents

Optimized geometry of silver-tyrosine systems **S2**

Molecular orbitals of Tyr^{-1} systems **S6**

Absorption spectra of SemiQ^{-1}-NC complexes **S7**

Molecular orbitals of Tyr^{-2}-Ag$_n^q$ and SemiQ^{-1}-Ag$_n^q$ systems **S8**

Infrared spectra **S9**

AIM and NBO analysis **S11**

Cartesian coordinates of Tyr^{-1} systems **S13**

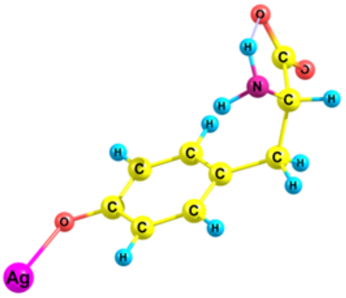

Ag^0

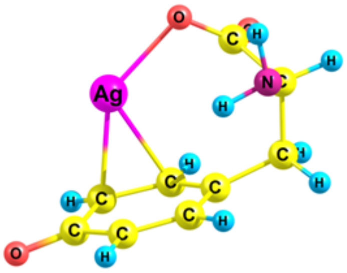

Ag^+

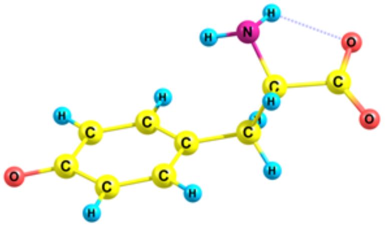

Tyr^{-2}

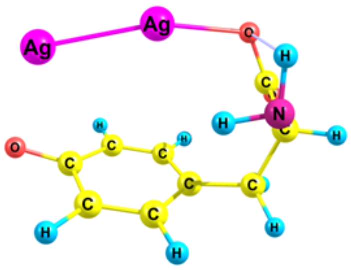

$Ag_2{}^0$

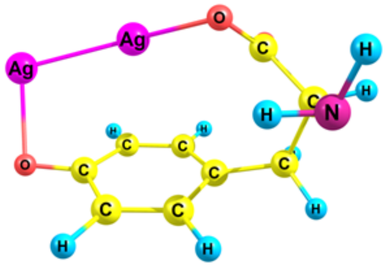

$Ag_2{}^+$

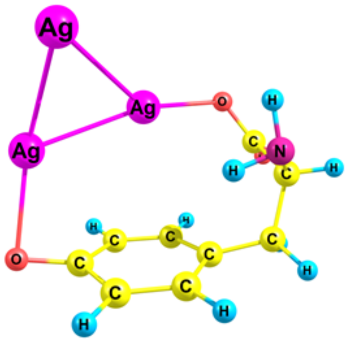

$Ag_3{}^0$ (D_{3h})

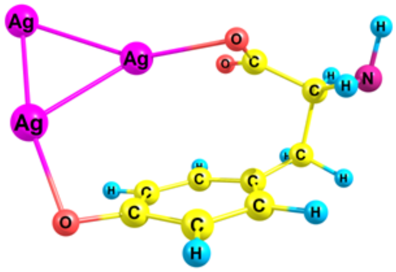

$Ag_3{}^+$ (D_{3h})

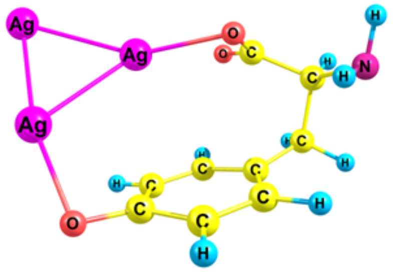

$Ag_3{}^{2+}$ (D_{3h})

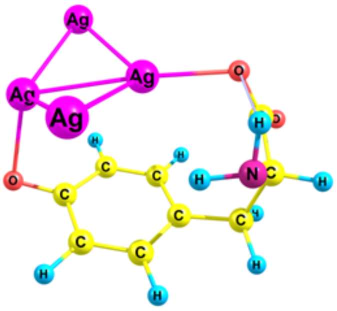

$Ag_4{}^0$ (D_{2h})

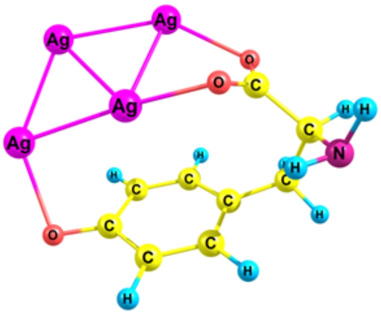

$Ag_4{}^+$ (D_{2h})

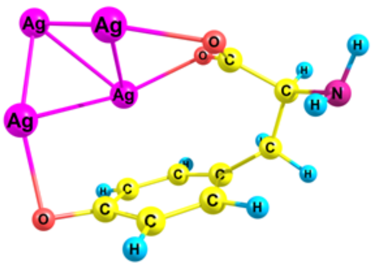

$Ag_4{}^{2+}$ (T_d)

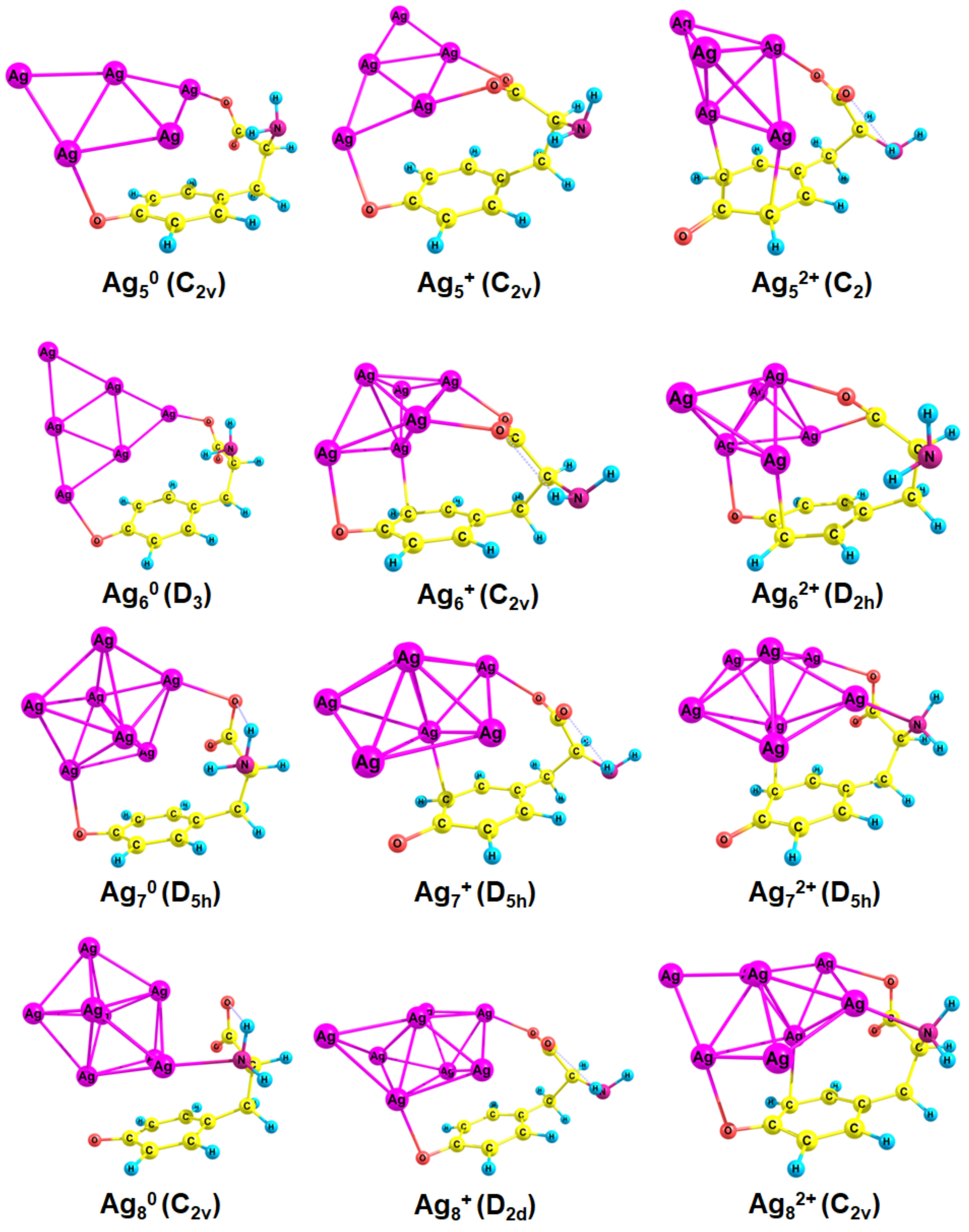

Figure S1. Established complexes of Ag_n^q (n = 1-8, q = 0-2) NCs and Tyr^{-2} (PBE-D3 optimization); point group of a cluster is written in brackets.

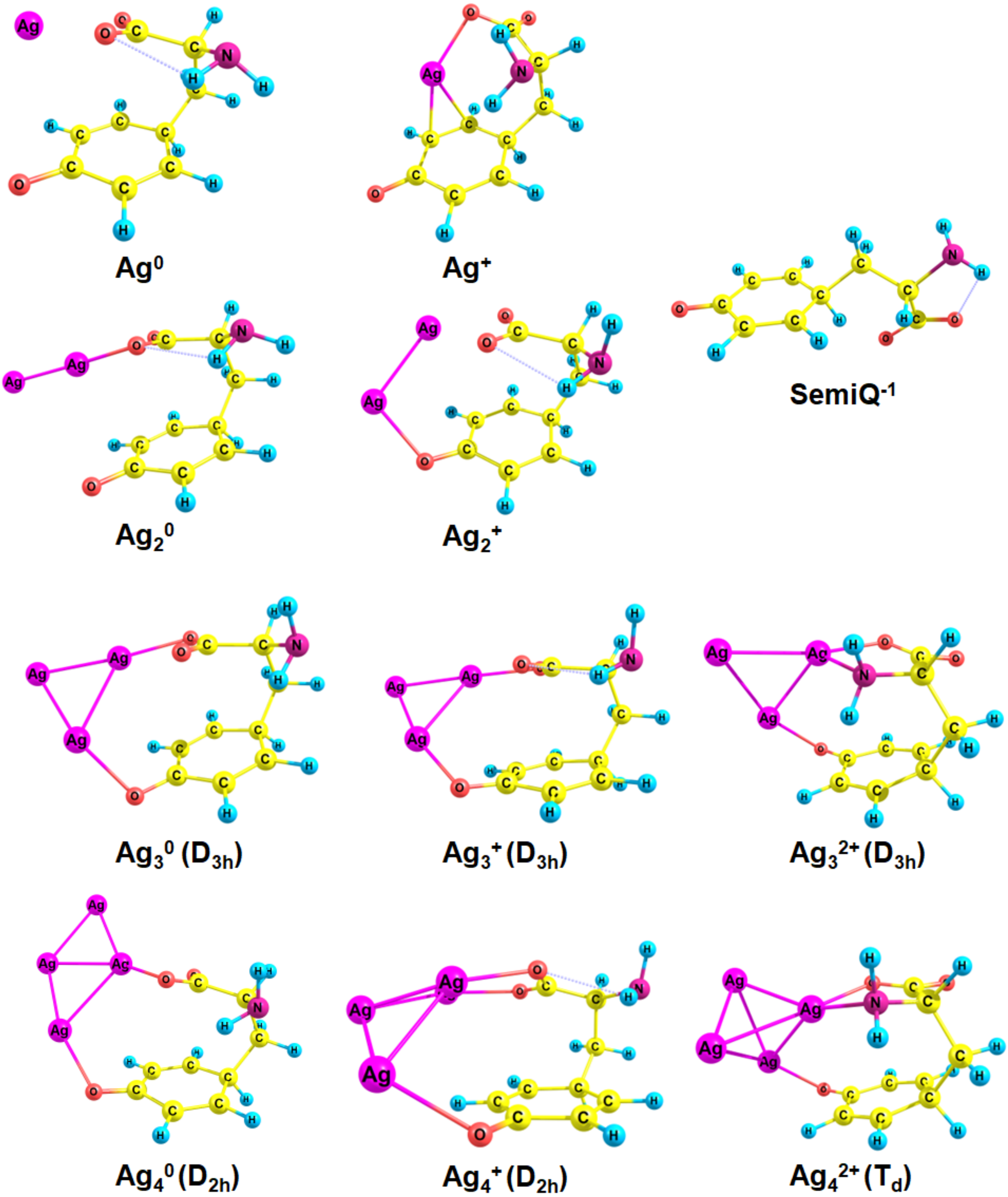
Ag^0
Ag^+
Ag_2^0
Ag_2^+
$SemiQ^{-1}$
Ag_3^0 (D_{3h})
Ag_3^+ (D_{3h})
Ag_3^{2+} (D_{3h})
Ag_4^0 (D_{2h})
Ag_4^+ (D_{2h})
Ag_4^{2+} (T_d)

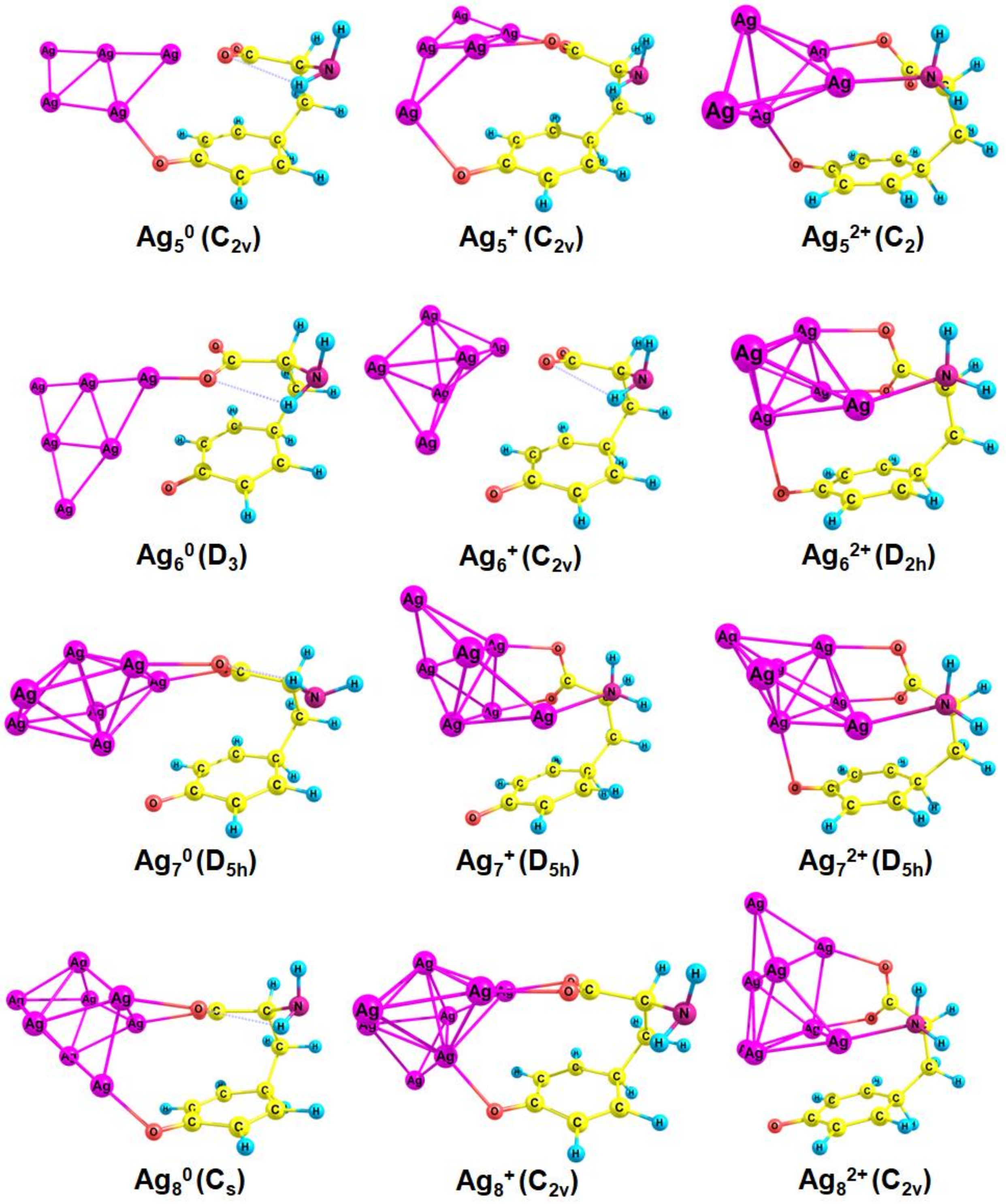

Figure S2. Established complexes of Ag_n^q (n = 1-8, q = 0-2) NCs and $SemiQ^{-1}$ (PBE-D3 optimization); point group of a cluster is written in brackets.

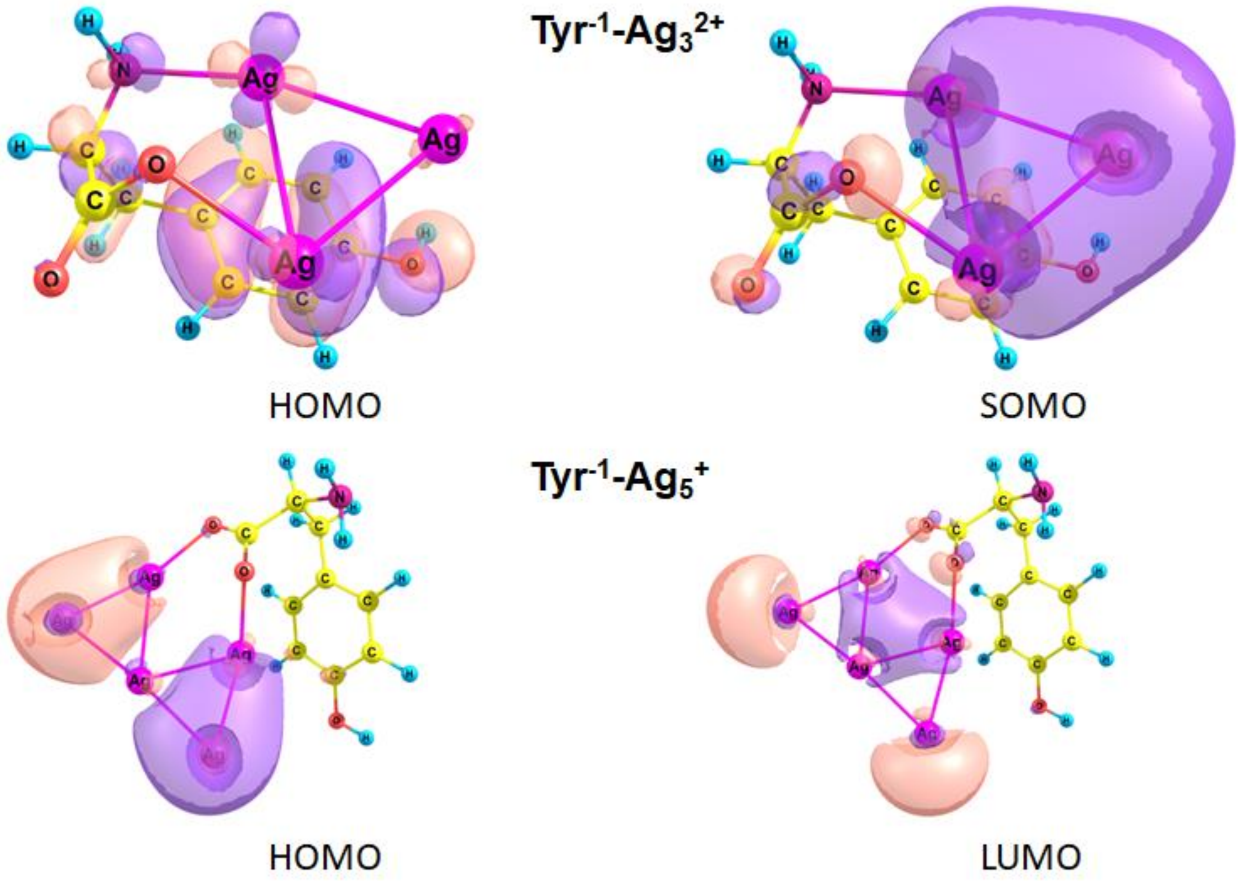

Figure S3. Molecular orbitals responsible for the main transitions in the UV-vis spectra of Tyr^{-1}-Ag_n^q complexes.

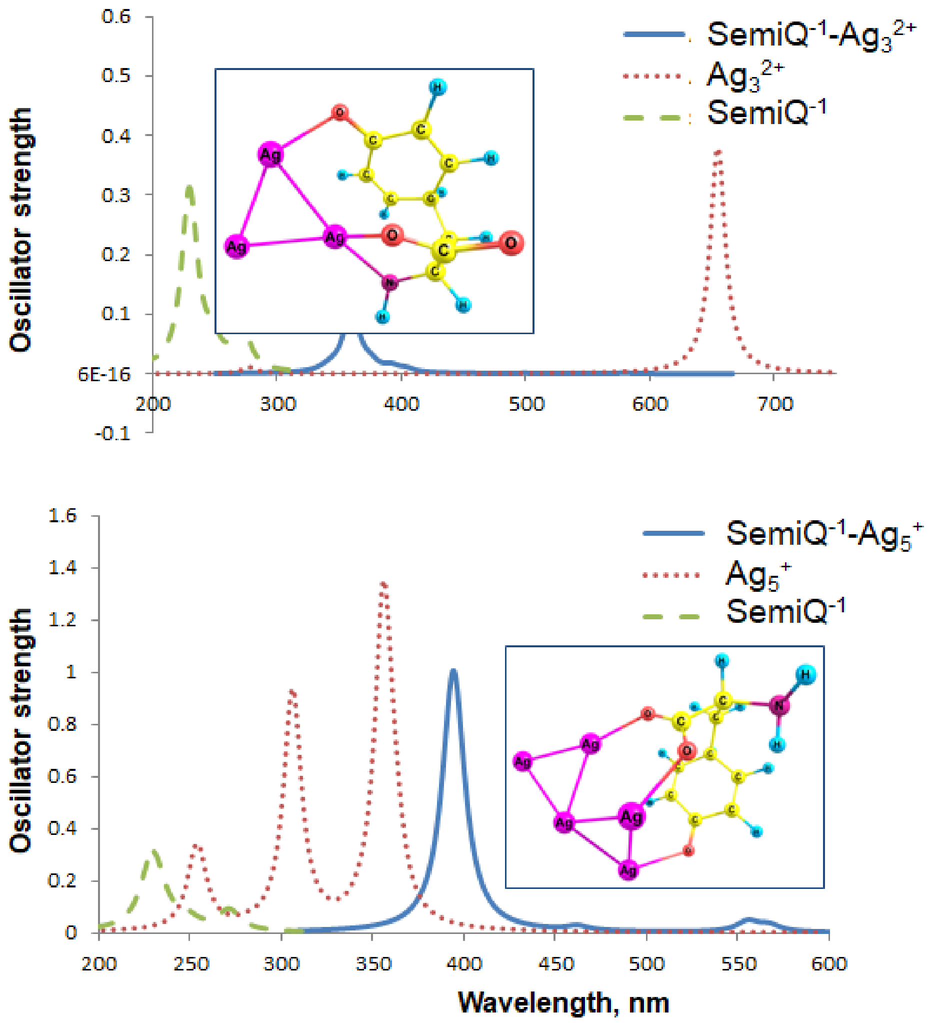

Figure S4. Absorption spectra of $SemiQ^{-1}$ complexes with Ag nanoclusters according to TDDFT M062X/def2-TZVP. Lorentzian broadening with a bandwidth at ½ height equal to 15 nm was used.

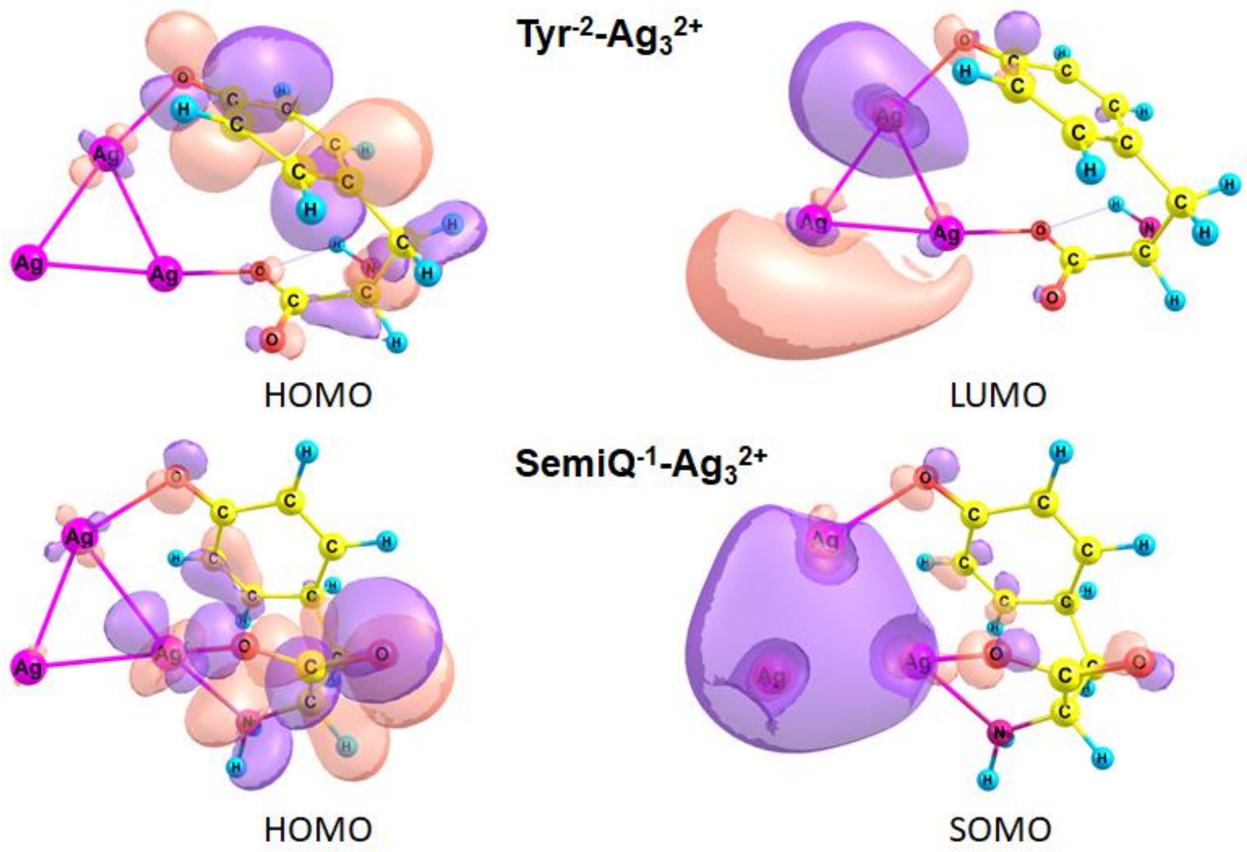

Figure S5. Molecular orbitals responsible for the main transitions in the absorption spectra of Tyr^{-2}-Ag_n^q and $SemiQ^{-1}$-Ag_n^q systems.

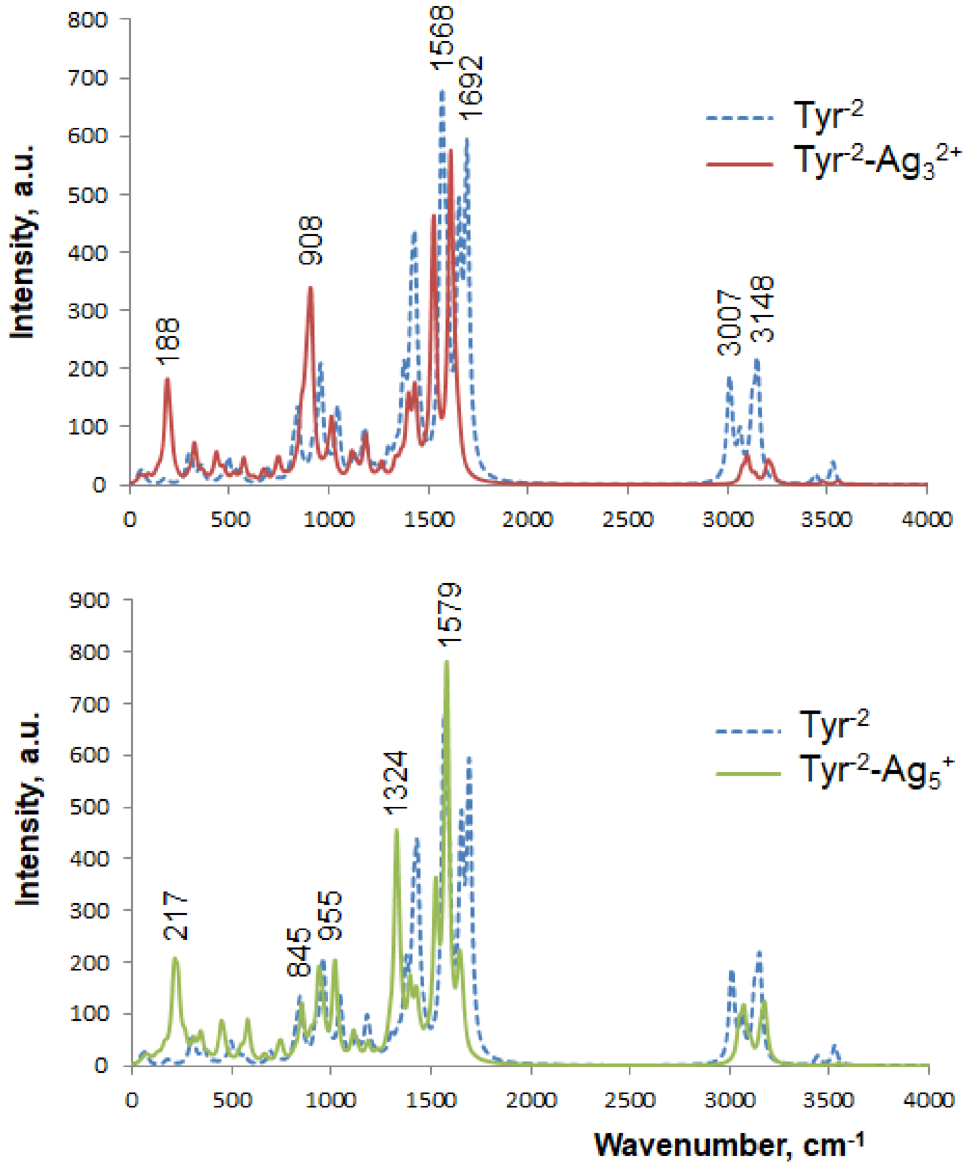

Figure S6. Infrared spectra of bare Tyr^{-2} and its complexes (Tyr^{-2}-Ag_3^{2+}, Tyr^{-2}-Ag_5^{+}) according to B3LYP-D3/6-31G(d,p),LANLTZ method. Lorentzian broadening with a bandwidth at ½ height equal to 30 cm^{-1} was used.

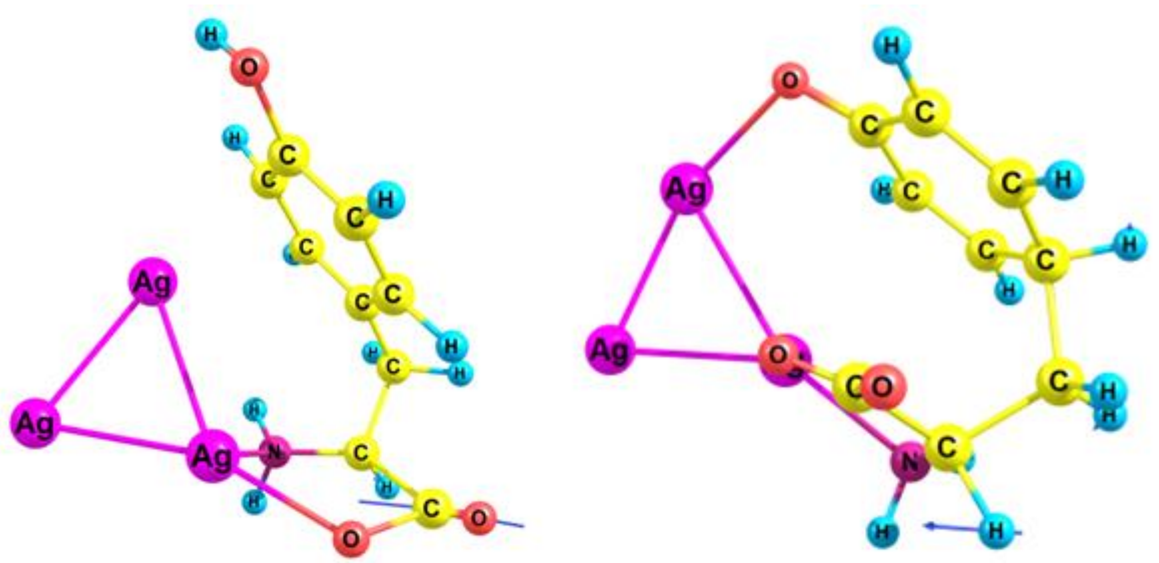

Figure S7. Geometry of Tyr^{-1}–Ag_3^{2+} complex with O-C-O asymmetric stretching (1698 cm^{-1}) on the left; b) $SemiQ^{-1}$–Ag_3^{2+} complex with C-H scissoring vibrations indicated (1324 cm^{-1}) on the right.

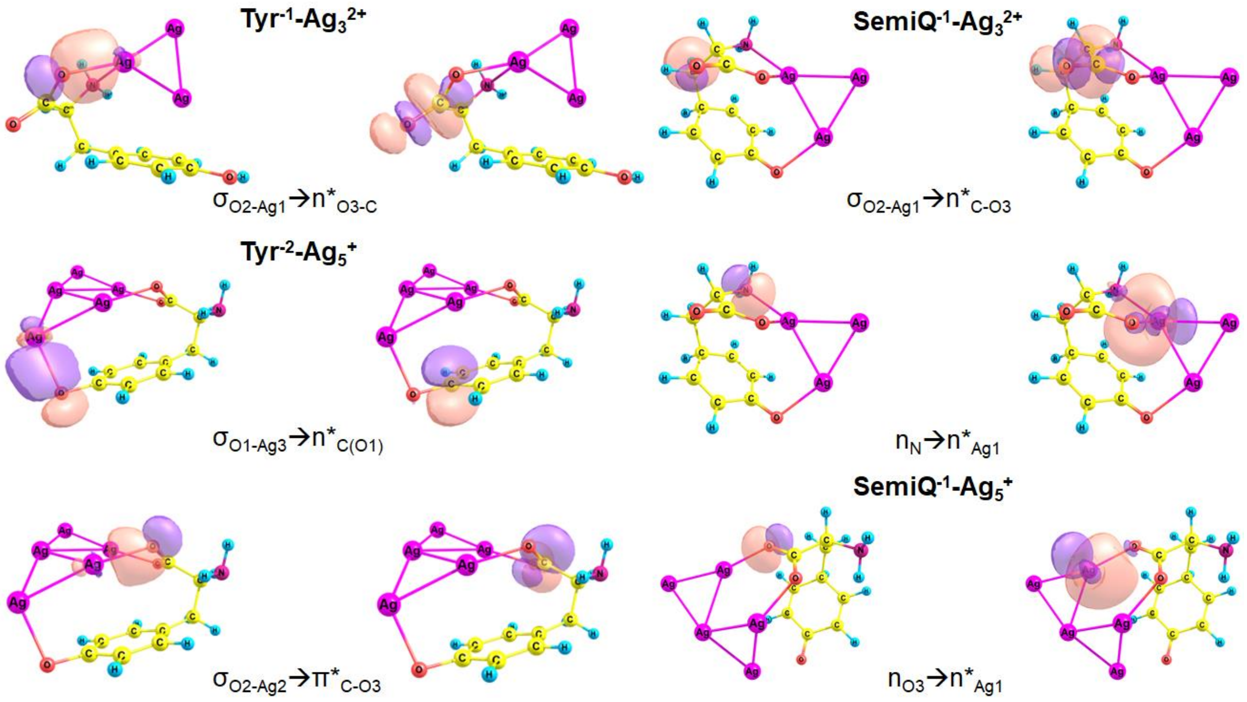

Figure S8. Selected NBO orbitals involved in charge transfer.

We have used several QTAIM parameters that are presented in Table S1: the density of all electrons ρ, the Laplacian of the electron density $\nabla^2\rho$, the Lagrangian kinetic energy G, the potential energy density V, and the energy density H.

A positive value of the Laplacian $\nabla^2\rho$ indicates depletion of the electronic charge along the bond, which is typical for electrostatic interactions. A negative value of $\nabla^2\rho$ demonstrates that the electronic charge is located between the nuclei, which is characteristic for covalent interactions. The electronic energy density term H is the sum of kinetic and potential components:

$H = G + V$ (eq. 1)

The virial theorem states that G and V are related to the Laplacian through the equation:

$\frac{1}{4}\nabla^2\rho = 2G + V$ (eq. 2)

If H is positive, then the accumulation of charge at this point is destabilizing. If H is below zero, then the accumulation of charge is stabilizing. The negative value of H shows that the bond is covalent.

The value of the potential energy V allows one to calculate the bond energy as follows:

$E_{bond} = - V * 627.5 / 2$ (eq. 3)

Table S1. Bond critical point (BCP) data (all in hartree except bond energy, which is in kcal mol^{-1}) according to QTAIM analysis.

**Complex**	**BCP**	**ρ**	**$\nabla^2\rho$**	**G**	**V**	**H**	**E_{bond}**
Tyr^{-1}-Ag_3^{2+}	Ag-N	0.0589	0.2501	0.0693	-0.0761	-0.0068	23.9
Tyr^{-1}-Ag_3^{2+}	Ag-O2	0.0532	0.2636	0.0686	-0.0714	-0.0027	22.4
Tyr^{-1}-Ag_5^{+}	Ag-O2	0.0468	0.2338	0.0596	-0.0607	-0.0011	19
Tyr^{-1}-Ag_5^{+}	Ag-O3	0.0579	0.2826	0.0748	-0.079	-0.0042	24.8
Tyr^{-2}-Ag_3^{2+}	Ag-O1	0.0485	0.226	0.0593	-0.0621	-0.0028	19.5
Tyr^{-2}-Ag_3^{2+}	Ag-O2	0.0513	0.2513	0.0656	-0.0684	-0.0028	21.5
Tyr^{-2}-Ag_5^{+}	Ag-O1	0.0509	0.2382	0.0629	-0.0663	-0.0034	20.8
Tyr^{-2}-Ag_5^{+}	Ag-O2	0.0553	0.2661	0.0702	-0.0739	-0.0037	23.2
Tyr^{-2}-Ag_5^{+}	Ag-O3	0.0512	0.2593	0.0668	-0.0688	-0.002	21.6

$SemiQ^{-1}$-Ag_3^{2+}	Ag-N	0.059	0.2509	0.0696	-0.0765	-0.0069	24
$SemiQ^{-1}$-Ag_3^{2+}	Ag-O1	0.0505	0.2524	0.065	-0.067	-0.0019	21
$SemiQ^{-1}$-Ag_3^{2+}	Ag-O2	0.0528	0.2565	0.0671	-0.07	-0.003	22
$SemiQ^{-1}$-Ag_5^{+}	Ag-O1	0.0329	0.1444	0.0363	-0.0364	-0.0002	11.4
$SemiQ^{-1}$-Ag_5^{+}	Ag-O2	0.0597	0.3063	0.0802	-0.0838	-0.0036	26.3
$SemiQ^{-1}$-Ag_5^{+}	Ag-O3	0.0602	0.3133	0.0818	-0.0853	-0.0035	26.8

Table S2. Calculated natural population analysis (NPA) charges and Wiberg bond indices of the Tyr-Ag_n^q systems (B3LYP-D3/6-31G(d,p),LANLTZ method), for atom numbering see Figure 3.

**Complex**	**Bond type**	$W_{Ag\text{-}X}$	$W_{Ag1\text{-}Ag2}$	q_X	q_{Agn}	$q_{cluster}$
Ag_3^{2+}	-	-	0.929	-	0.67	2
Tyr^{-1}-Ag_3^{2+}	Ag1-N	0.41	0.015	-0.376	0.192	0.962
Tyr^{-1}-Ag_3^{2+}	Ag1-O2	0.611	0.015	-0.273	0.192	0.962
$SemiQ^{-1}$-Ag_3^{2+}	Ag1-N	0.458	0.7	-0.375	0.226	1.129
$SemiQ^{-1}$-Ag_3^{2+}	Ag2-O1	0.504	0.7	-0.211	0.369	1.129
$SemiQ^{-1}$-Ag_3^{2+}	Ag1-O2	0.58	0.7	-0.269	0.226	1.129
Tyr^{-2}-Ag_3^{2+}	Ag2-O1	0.565	0.909	-0.271	0.196	0.716
Tyr^{-2}-Ag_3^{2+}	Ag1-O2	0.474	0.909	-0.261	0.122	0.716
Ag_5^{+}	-	-	1.125	-	0.348	1
Tyr^{-1}-Ag_5^{+}	Ag1-O2	0.473	0.612	-0.25	-0.018	0.009
Tyr^{-1}-Ag_5^{+}	Ag2-O3	0.512	0.612	-0.233	-0.048	0.009
$SemiQ^{-1}$-Ag_5^{+}	Ag3-O1	0.333	0.708	-0.184	0.197	0.057
$SemiQ^{-1}$-Ag_5^{+}	Ag2-O2	0.491	0.708	-0.243	-0.033	0.057
$SemiQ^{-1}$-Ag_5^{+}	Ag1-O3	0.498	0.708	-0.237	-0.037	0.057
Tyr^{-2}-Ag_5^{+}	Ag3-O1	0.546	0.674	-0.278	0.024	-0.393
Tyr^{-2}-Ag_5^{+}	Ag2-O2	0.453	0.674	-0.254	-0.011	-0.393
Tyr^{-2}-Ag_5^{+}	Ag1-O3	0.501	0.674	-0.234	-0.1	-0.393

Cartesian coordinates of isolated Tyr^{-1} and most stable Tyr^{-1}-bound silver nanoclusters optimized using PBE-D3/6-31G(d,p),LANLTZ method:

Tyr^{-1}

N	-1.894680000	1.512730000	1.029236000
C	-2.175103000	0.726049000	-0.177848000
C	-2.426058000	-0.794210000	0.098021000
O	-2.494768000	-1.521372000	-0.927041000
H	-2.357195000	0.972933000	1.765556000
H	-0.907005000	1.371440000	1.243217000
H	-3.109247000	1.100045000	-0.618264000
C	-1.079822000	0.929443000	-1.227888000
C	0.275964000	0.508099000	-0.733973000
H	-1.040719000	1.985993000	-1.517080000
H	-1.361251000	0.334346000	-2.098899000
C	2.788709000	-0.278038000	0.255723000
C	1.255480000	1.450497000	-0.404268000
C	0.581915000	-0.846978000	-0.551456000
C	1.827033000	-1.234232000	-0.064430000
C	2.504725000	1.071710000	0.086124000
H	1.031803000	2.506579000	-0.527479000
H	-0.179687000	-1.586730000	-0.781627000
H	2.046909000	-2.290382000	0.076884000
H	3.258379000	1.808108000	0.343432000
O	4.034672000	-0.620396000	0.740506000
H	4.038747000	-1.579464000	0.854862000
O	-2.556055000	-1.114117000	1.304534000

Ag^{+}-Tyr^{-1}

N	-1.878143000	-1.702787000	-0.893099000
C	-2.191710000	-0.780107000	0.198328000
C	-2.415736000	0.684595000	-0.241344000
O	-2.447873000	0.927974000	-1.469760000
H	-0.933700000	-1.474716000	-1.203765000
H	-2.451591000	-1.393770000	-1.677696000
H	-3.136230000	-1.101762000	0.654818000
C	-1.115002000	-0.860203000	1.286161000
C	0.244382000	-0.487989000	0.764455000
H	-1.084396000	-1.881923000	1.678481000
H	-1.409026000	-0.182400000	2.089469000
C	2.758412000	0.201915000	-0.287629000
C	0.592375000	0.852668000	0.545037000
C	1.182496000	-1.470225000	0.439208000
C	2.432526000	-1.135331000	-0.082133000
C	1.835397000	1.196631000	0.025150000
H	-0.134460000	1.626257000	0.772523000
H	0.928911000	-2.515610000	0.589475000
H	3.150762000	-1.914823000	-0.326152000
H	2.097104000	2.234019000	-0.151183000
O	3.977888000	0.593861000	-0.793149000
H	4.474038000	-0.207184000	-1.004747000
O	-2.570336000	1.524598000	0.695188000

Ag	-2.787964000	3.423237000	-0.728530000

$Ag\text{-}Tyr^{-1}$

N	-1.956816000	-1.748864000	-0.862454000
C	-2.247849000	-0.801074000	0.221540000
C	-2.483219000	0.666544000	-0.243223000
O	-2.478166000	0.902022000	-1.491872000
H	-0.987068000	-1.565955000	-1.156273000
H	-2.505073000	-1.428873000	-1.671571000
H	-3.191703000	-1.110775000	0.712916000
C	-1.138961000	-0.849207000	1.301767000
C	0.220209000	-0.441860000	0.774572000
H	-1.091091000	-1.875619000	1.702295000
H	-1.449494000	-0.177213000	2.116638000
C	2.737535000	0.324252000	-0.287489000
C	0.570384000	0.919166000	0.635890000
C	1.163508000	-1.405400000	0.367287000
C	2.410459000	-1.037146000	-0.158777000
C	1.809731000	1.304320000	0.115033000
H	-0.152344000	1.684248000	0.935837000
H	0.921488000	-2.469146000	0.468635000
H	3.130134000	-1.803318000	-0.466858000
H	2.075633000	2.360895000	0.015299000
O	3.938079000	0.755131000	-0.789647000
H	4.471639000	-0.027629000	-1.028918000
O	-2.680883000	1.532218000	0.676516000
Ag	-2.623561000	3.413331000	-0.957212000

$Ag_2^{+}\text{-}Tyr^{-1}$

N	-3.425061000	-1.650720000	-0.826072000
C	-3.401866000	-0.379966000	-0.122842000
C	-2.910195000	0.801528000	-0.910891000
O	-2.267550000	0.557716000	-1.977740000
H	-2.651444000	-1.629164000	-1.491585000
H	-4.259616000	-1.661302000	-1.408758000
H	-4.394323000	-0.164609000	0.286475000
C	-2.423506000	-0.453289000	1.115930000
C	-1.022904000	-0.114532000	0.672351000
H	-2.477162000	-1.452609000	1.550894000
H	-2.739526000	0.273042000	1.864837000
C	1.362754000	0.739969000	-0.558157000
C	-0.517515000	1.174842000	0.889272000
C	-0.259549000	-0.990036000	-0.107068000
C	0.918751000	-0.568367000	-0.721309000
C	0.658044000	1.609524000	0.269382000
H	-1.061571000	1.852387000	1.536960000
H	-0.603047000	-2.008412000	-0.258627000
H	1.489265000	-1.260065000	-1.335912000
H	1.077692000	2.585995000	0.492175000
O	2.496764000	1.227111000	-1.161089000
H	2.889628000	0.513069000	-1.681913000
O	-2.988054000	1.945970000	-0.381572000

Ag	-1.084624000	2.423094000	-1.597720000
Ag	1.019224000	4.083757000	-2.001568000

Ag_2-Tyr^{-1}

N	-1.899960000	-1.588333000	-0.872928000
C	-2.187908000	-0.607935000	0.178668000
C	-2.268170000	0.900624000	-0.228342000
O	-1.838747000	1.260400000	-1.391150000
H	-0.914685000	-1.493335000	-1.154235000
H	-2.422056000	-1.314285000	-1.710449000
H	-3.190000000	-0.836398000	0.580826000
C	-1.190656000	-0.774981000	1.353811000
C	0.213978000	-0.442295000	0.927615000
H	-1.248029000	-1.815696000	1.710398000
H	-1.524659000	-0.105686000	2.158410000
C	2.631335000	0.268686000	-0.341171000
C	0.664668000	0.887012000	0.924690000
C	1.046553000	-1.418410000	0.360571000
C	2.241077000	-1.072809000	-0.272122000
C	1.862244000	1.241697000	0.302733000
H	0.022850000	1.663193000	1.345988000
H	0.721517000	-2.462125000	0.364607000
H	2.851663000	-1.829815000	-0.766626000
H	2.175220000	2.284873000	0.238631000
O	3.735732000	0.652053000	-1.078501000
H	3.540218000	0.373274000	-2.009596000
O	-2.784704000	1.662208000	0.608203000
Ag	-0.086162000	0.581813000	-2.476589000
Ag	1.958573000	-0.068540000	-3.968643000

Ag_3^{2+}-Tyr^{-1}

N	-1.901454000	-1.117104000	-1.226478000
C	-2.194600000	-0.541710000	0.090043000
C	-2.389636000	1.004560000	0.003005000
O	-2.189954000	1.609748000	-1.122949000
H	-1.266138000	-1.919813000	-1.193643000
H	-2.744516000	-1.384924000	-1.744170000
H	-3.154506000	-0.946832000	0.452825000
C	-1.096027000	-0.887787000	1.120263000
C	0.291257000	-0.451409000	0.705102000
H	-1.113302000	-1.974473000	1.299437000
H	-1.392400000	-0.389816000	2.058428000
C	2.886176000	0.406195000	-0.124345000
C	0.674272000	0.911281000	0.757786000
C	1.234357000	-1.382912000	0.231493000
C	2.526282000	-0.972909000	-0.214506000
C	1.934220000	1.341886000	0.341327000
H	-0.031284000	1.648629000	1.150449000
H	0.998023000	-2.451986000	0.228936000
H	3.295338000	-1.724580000	-0.429441000
H	2.219053000	2.394229000	0.410239000
O	4.116254000	0.867481000	-0.439767000

H	4.693648000	0.121426000	-0.700935000
O	-2.725921000	1.601941000	1.044748000
Ag	-0.937280000	0.479627000	-2.539202000
Ag	1.527388000	-0.774794000	-2.344708000
Ag	0.374914000	-0.459604000	-4.736703000

Ag_3^+-Tyr^{-1}

N	2.709052000	1.577674000	-0.683194000
C	2.507281000	0.892522000	0.600017000
C	1.106448000	1.249207000	1.195754000
C	2.605873000	-0.645947000	0.496620000
C	-0.738103000	-0.484281000	1.010285000
C	-0.047996000	0.613993000	0.457203000
O	2.200523000	-1.184325000	-0.582763000
C	-1.738635000	-1.181810000	0.277965000
O	3.027948000	-1.283722000	1.530943000
C	-0.432073000	1.044329000	-0.831381000
C	-2.078262000	-0.737549000	-1.027902000
C	-1.417335000	0.381222000	-1.571516000
O	-3.071424000	-1.401843000	-1.677469000
H	2.217817000	1.012391000	-1.389377000
H	3.699867000	1.479521000	-0.936226000
H	3.263595000	1.251376000	1.315967000
H	1.087985000	0.932574000	2.251595000
H	1.029819000	2.349636000	1.167095000
H	-0.523459000	-0.798283000	2.037181000
H	-2.364666000	-1.937764000	0.763915000
H	0.060947000	1.916580000	-1.270420000
H	-1.693999000	0.739749000	-2.567681000
H	-3.202260000	-0.996196000	-2.557302000
Ag	2.461059000	-3.437135000	1.046791000
Ag	0.738942000	-5.294317000	0.178776000
Ag	0.128628000	-2.661640000	-0.142308000

Ag_3-Tyr^{-1}

N	-2.151152000	-1.470580000	-0.923819000
C	-2.321111000	-0.587933000	0.236797000
C	-2.516719000	0.932680000	-0.050168000
O	-2.393585000	1.361536000	-1.272284000
H	-1.172625000	-1.415733000	-1.250236000
H	-2.697091000	-1.081089000	-1.701871000
H	-3.252817000	-0.882874000	0.756769000
C	-1.167816000	-0.793261000	1.254409000
C	0.190270000	-0.473942000	0.673264000
H	-1.189875000	-1.839852000	1.600002000
H	-1.385627000	-0.143040000	2.116394000
C	2.663369000	0.149577000	-0.573864000
C	0.609999000	0.866049000	0.507622000
C	1.052418000	-1.489035000	0.219113000
C	2.275593000	-1.190984000	-0.402649000
C	1.830171000	1.181490000	-0.104697000
H	-0.031293000	1.674213000	0.873707000

H	0.761805000	-2.537526000	0.342779000
H	2.930914000	-1.997614000	-0.748049000
H	2.143735000	2.221055000	-0.230270000
O	3.828793000	0.509171000	-1.197226000
H	4.272028000	-0.300879000	-1.516848000
O	-2.832899000	1.654673000	0.923457000
Ag	-0.532809000	0.850847000	-2.345967000
Ag	1.627173000	1.376161000	-3.831824000
Ag	0.837856000	-1.278342000	-3.387508000

Ag_4^{2+}-Tyr^{-1}

N	-2.424138000	-1.070307000	-0.958290000
C	-2.249868000	-0.901817000	0.504895000
C	-2.736687000	0.501107000	0.965533000
O	-3.611642000	1.079689000	0.181507000
H	-2.367440000	-2.062244000	-1.212538000
H	-3.370930000	-0.746482000	-1.190217000
H	-2.910384000	-1.620879000	1.026930000
C	-0.810384000	-1.223726000	0.969376000
C	0.289902000	-0.380375000	0.366409000
H	-0.609841000	-2.286615000	0.752261000
H	-0.809821000	-1.102901000	2.063266000
C	2.465409000	1.128466000	-0.734577000
C	0.666818000	0.859975000	0.927176000
C	1.006456000	-0.827729000	-0.773571000
C	2.062781000	-0.087697000	-1.332561000
C	1.737686000	1.625697000	0.387472000
H	0.140379000	1.227266000	1.812068000
H	0.772743000	-1.809264000	-1.200265000
H	2.610756000	-0.478898000	-2.194878000
H	2.114070000	2.505443000	0.919761000
O	3.532177000	1.844973000	-1.157378000
H	3.932819000	1.405625000	-1.934184000
O	-2.334330000	0.936736000	2.060205000
Ag	-2.637899000	2.413809000	-1.259361000
Ag	-1.086017000	2.798855000	-3.489968000
Ag	0.130970000	2.613528000	-1.068028000
Ag	-1.079075000	0.292877000	-2.182414000

Ag_4^{+}-Tyr^{-1}

N	2.690058000	1.559355000	-0.696101000
C	2.484171000	0.899747000	0.591811000
Ag	2.219191000	-3.468957000	1.407910000
C	1.110853000	1.306323000	1.183390000
C	2.484418000	-0.632985000	0.523203000
C	-0.640334000	-0.503248000	1.015827000
C	-0.026956000	0.633422000	0.463516000
O	2.246621000	-1.145720000	-0.622988000
C	-1.619410000	-1.216703000	0.315421000
O	2.620373000	-1.260738000	1.621297000
C	-0.447240000	1.060097000	-0.805399000
C	-2.006137000	-0.785852000	-0.960565000

C	-1.419687000	0.354351000	-1.520790000
O	-2.967148000	-1.535221000	-1.609860000
H	2.262516000	0.955897000	-1.395194000
H	3.682899000	1.534302000	-0.910868000
H	3.256903000	1.224953000	1.291026000
H	1.089272000	1.026962000	2.238324000
H	1.035161000	2.392484000	1.106917000
H	-0.346946000	-0.839255000	2.004609000
H	-2.118133000	-2.069561000	0.758538000
H	0.004549000	1.942991000	-1.242833000
H	-1.733072000	0.700979000	-2.501104000
H	-3.139397000	-1.130052000	-2.471039000
Ag	0.516658000	-5.242887000	0.050166000
Ag	0.595387000	-2.630032000	-0.906430000
Ag	-1.434982000	-4.300701000	-1.716834000

Ag_4-Tyr^{-1}

N	-1.816139000	-2.206452000	-0.569087000
C	-2.197064000	-1.125392000	0.351043000
C	-2.514416000	0.210067000	-0.361301000
O	-2.091846000	0.371938000	-1.544478000
H	-1.281992000	-1.751998000	-1.322796000
H	-2.671352000	-2.521650000	-1.042254000
H	-3.092270000	-1.441220000	0.913296000
C	-1.075216000	-0.865656000	1.400301000
C	0.157169000	-0.197966000	0.834752000
H	-0.815722000	-1.845178000	1.835975000
H	-1.494331000	-0.236937000	2.201715000
C	2.451553000	1.084560000	-0.261138000
C	0.432355000	1.154172000	1.112268000
C	1.058685000	-0.892170000	-0.000567000
C	2.183526000	-0.266117000	-0.552265000
C	1.561224000	1.809192000	0.567959000
H	-0.233946000	1.714933000	1.775508000
H	0.873475000	-1.947572000	-0.220393000
H	2.875217000	-0.830933000	-1.187092000
H	1.834439000	2.819250000	0.890188000
O	3.554021000	1.740851000	-0.728762000
H	4.067621000	1.128800000	-1.291639000
O	-3.138839000	1.106089000	0.320226000
Ag	-2.892084000	2.836648000	-1.190918000
Ag	-1.409560000	4.689355000	-2.463806000
Ag	0.026407000	2.863808000	-1.170737000
Ag	-4.230834000	4.803881000	-2.508437000

Ag_5^{2+}-Tyr^{-1}

N	-2.257993000	-1.277976000	-0.971461000
C	-2.227624000	-0.913428000	0.471420000
C	-2.738686000	0.546895000	0.564974000
O	-3.719405000	0.820035000	-0.235695000
H	-1.966605000	-2.252450000	-1.106150000
H	-3.228636000	-1.200326000	-1.294241000

H	-2.933982000	-1.553614000	1.033463000
C	-0.829707000	-1.141015000	1.098128000
C	0.315008000	-0.390224000	0.459054000
H	-0.626918000	-2.225750000	1.050505000
H	-0.907404000	-0.872342000	2.161800000
C	2.397212000	1.092151000	-0.834006000
C	0.828742000	0.801710000	1.032327000
C	0.916558000	-0.843178000	-0.739169000
C	1.933810000	-0.118508000	-1.385327000
C	1.852218000	1.551983000	0.392913000
H	0.473257000	1.120210000	2.016379000
H	0.612232000	-1.805383000	-1.162548000
H	2.380061000	-0.504715000	-2.306295000
H	2.297678000	2.420853000	0.886236000
O	3.372587000	1.843589000	-1.401729000
H	3.677693000	1.409754000	-2.223286000
O	-2.158355000	1.365462000	1.330603000
Ag	-2.956109000	2.637875000	-1.347906000
Ag	-0.701809000	2.916451000	-2.998914000
Ag	-0.394673000	2.483134000	-0.237944000
Ag	-1.170107000	0.341430000	-2.179102000
Ag	-1.436873000	4.927761000	-1.088987000

Ag_5^+-Tyr^{-1}

N	-2.437632000	-1.747208000	-0.526223000
C	-2.460456000	-0.672083000	0.466236000
C	-2.485282000	0.752303000	-0.134844000
O	-2.032919000	0.886047000	-1.322921000
H	-1.762942000	-1.469168000	-1.250033000
H	-3.340390000	-1.747959000	-1.014713000
H	-3.346220000	-0.799932000	1.109927000
C	-1.195797000	-0.750503000	1.386261000
C	0.062348000	-0.255586000	0.710587000
H	-1.094057000	-1.806643000	1.685143000
H	-1.389160000	-0.154235000	2.292074000
C	2.276506000	0.798362000	-0.732673000
C	0.525921000	1.055955000	0.946237000
C	0.754048000	-1.034374000	-0.240509000
C	1.841167000	-0.520367000	-0.962708000
C	1.617372000	1.591265000	0.235033000
H	0.027000000	1.679557000	1.695067000
H	0.429378000	-2.061772000	-0.431808000
H	2.357363000	-1.142277000	-1.702244000
H	1.993484000	2.593833000	0.453597000
O	3.317857000	1.372855000	-1.408819000
H	3.646591000	0.743775000	-2.081524000
O	-2.877711000	1.703648000	0.625259000
Ag	-0.290167000	2.327923000	-1.555882000
Ag	-0.695494000	5.016830000	-1.910596000
Ag	1.600536000	3.820608000	-2.748282000
Ag	-2.878038000	6.306491000	-0.903190000
Ag	-2.592218000	3.715166000	-0.310785000

Ag_5-Tyr^{-1}

N	-2.205313000	-1.459580000	-0.991925000
C	-2.297332000	-0.554341000	0.160513000
C	-2.438933000	0.974925000	-0.124775000
O	-2.474286000	1.388673000	-1.354472000
H	-1.269388000	-1.369174000	-1.419271000
H	-2.848927000	-1.118829000	-1.717216000
H	-3.227779000	-0.809011000	0.703789000
C	-1.137855000	-0.812255000	1.154114000
C	0.232286000	-0.473808000	0.610756000
H	-1.168172000	-1.872818000	1.452675000
H	-1.343269000	-0.199278000	2.044928000
C	2.826130000	0.195990000	-0.365457000
C	0.705474000	0.858733000	0.622852000
C	1.080158000	-1.464410000	0.081409000
C	2.367994000	-1.147501000	-0.424359000
C	1.974089000	1.197219000	0.141703000
H	0.058038000	1.641485000	1.028293000
H	0.760989000	-2.511628000	0.079116000
H	3.056777000	-1.947116000	-0.718723000
H	2.332236000	2.229473000	0.176644000
O	4.071829000	0.567491000	-0.779817000
H	4.545110000	-0.222355000	-1.109614000
O	-2.574853000	1.719277000	0.875340000
Ag	-0.975675000	0.714563000	-2.842555000
Ag	0.479161000	0.253092000	-5.056157000
Ag	1.375052000	-0.704092000	-2.655613000
Ag	2.916586000	-1.116459000	-4.966859000
Ag	1.940547000	-0.153504000	-7.337668000

Ag_6^{2+}-Tyr^{-1}

N	-2.298476000	-1.475383000	-1.467627000
C	-2.715385000	-0.303729000	-0.657113000
C	-3.014205000	0.869701000	-1.617883000
O	-3.314488000	0.566970000	-2.822509000
H	-2.200960000	-2.302810000	-0.868763000
H	-3.043075000	-1.677826000	-2.143554000
H	-3.658638000	-0.531032000	-0.122870000
C	-1.667243000	0.040651000	0.430631000
C	-0.275261000	0.348070000	-0.071543000
H	-1.622143000	-0.813764000	1.127439000
H	-2.063999000	0.900024000	0.990668000
C	2.373632000	0.892188000	-1.010994000
C	0.141058000	1.669131000	-0.337874000
C	0.674082000	-0.686260000	-0.267827000
C	1.971624000	-0.431368000	-0.734392000
C	1.446577000	1.955351000	-0.824270000
H	-0.544796000	2.497428000	-0.140700000
H	0.404853000	-1.716187000	-0.011346000
H	2.682075000	-1.253612000	-0.862143000
H	1.801599000	2.990254000	-0.884934000

O	3.622793000	1.216416000	-1.422775000
H	4.158219000	0.401887000	-1.507111000
O	-2.933328000	2.049062000	-1.133483000
Ag	-1.696652000	1.093871000	-4.486062000
Ag	0.820693000	-0.064533000	-4.967111000
Ag	-0.565303000	3.688068000	-4.950545000
Ag	0.423822000	1.916435000	-2.999330000
Ag	-2.101418000	3.368419000	-2.790302000
Ag	-0.538473000	-0.938828000	-2.819229000

Ag_6^+-Tyr^{-1}

N	2.690792000	1.000019000	-0.886515000
C	2.495505000	0.740154000	0.547175000
Ag	1.654569000	-3.384167000	1.697132000
C	1.115722000	1.255382000	1.029204000
C	2.754531000	-0.736151000	0.961483000
C	-0.806938000	-0.413925000	1.101260000
C	-0.090232000	0.583907000	0.408430000
O	3.450616000	-1.455911000	0.151030000
C	-1.961787000	-1.028677000	0.551545000
O	2.278253000	-1.140616000	2.071616000
C	-0.568806000	0.958116000	-0.867601000
C	-2.441743000	-0.607075000	-0.718070000
C	-1.711598000	0.365169000	-1.444737000
O	-3.595404000	-1.157027000	-1.167817000
H	2.012297000	0.423171000	-1.406602000
H	3.593967000	0.580814000	-1.140776000
H	3.250083000	1.337082000	1.094376000
H	1.075500000	1.132388000	2.121629000
H	1.081266000	2.335903000	0.809005000
H	-0.485136000	-0.704337000	2.104409000
H	-2.587353000	-1.687680000	1.164686000
H	-0.061441000	1.754794000	-1.420117000
H	-2.094684000	0.729767000	-2.403284000
H	-3.822471000	-0.772145000	-2.037859000
Ag	0.943592000	-5.298943000	-0.118763000
Ag	2.097063000	-2.750957000	-1.145162000
Ag	0.265849000	-4.195667000	-2.625824000
Ag	-0.439562000	-2.871810000	-0.078788000
Ag	0.084908000	-1.412876000	-2.501118000

Ag_6-Tyr^{-1}

N	-1.882004000	-1.991977000	-0.698246000
C	-2.234001000	-0.965315000	0.289105000
C	-2.373975000	0.464409000	-0.292154000
O	-1.771398000	0.708482000	-1.387055000
H	-1.143514000	-1.579708000	-1.284702000
H	-2.677784000	-2.088965000	-1.340548000
H	-3.191298000	-1.243926000	0.761407000
C	-1.157108000	-0.907898000	1.417394000
C	0.162094000	-0.363845000	0.921563000
H	-1.038130000	-1.932961000	1.805114000

H	-1.544909000	-0.274058000	2.230757000
C	2.493147000	0.724092000	-0.242369000
C	0.474349000	1.002953000	1.066245000
C	1.069517000	-1.178538000	0.216628000
C	2.226730000	-0.646050000	-0.370452000
C	1.629757000	1.553281000	0.494873000
H	-0.209570000	1.657029000	1.616581000
H	0.855001000	-2.245723000	0.104150000
H	2.912181000	-1.289668000	-0.932309000
H	1.868681000	2.613152000	0.615754000
O	3.582508000	1.329420000	-0.855563000
H	4.107932000	0.643230000	-1.314895000
O	-3.038510000	1.299641000	0.420038000
Ag	-2.703222000	3.420416000	-0.381505000
Ag	-1.617949000	5.368229000	-1.824173000
Ag	-0.410093000	2.722062000	-1.728910000
Ag	0.679217000	4.771465000	-3.317207000
Ag	-0.564571000	7.135837000	-3.685415000
Ag	2.217955000	2.725678000	-2.554502000

Ag_7^{2+}-Tyr^{-1}

N	-2.292634000	-1.250714000	-1.656584000
C	-2.621891000	-0.049927000	-0.843409000
C	-2.822237000	1.152356000	-1.790210000
O	-3.080191000	0.870290000	-3.021132000
H	-2.050072000	-2.034619000	-1.040839000
H	-3.124627000	-1.541444000	-2.180803000
H	-3.573383000	-0.205466000	-0.299423000
C	-1.539060000	0.217074000	0.227189000
C	-0.129354000	0.433364000	-0.276277000
H	-1.544628000	-0.638271000	0.924263000
H	-1.870894000	1.101154000	0.791489000
C	2.592205000	0.838766000	-1.059808000
C	0.356347000	1.732247000	-0.560624000
C	0.776312000	-0.642593000	-0.401700000
C	2.107663000	-0.457754000	-0.796314000
C	1.705049000	1.949260000	-0.963853000
H	-0.298204000	2.594516000	-0.407028000
H	0.440099000	-1.656060000	-0.162451000
H	2.784322000	-1.314722000	-0.865962000
H	2.112190000	2.964698000	-1.012760000
O	3.881886000	1.101419000	-1.379162000
H	4.390256000	0.265627000	-1.391554000
O	-2.668302000	2.327316000	-1.343839000
Ag	-0.419338000	1.194074000	-5.473085000
Ag	1.615758000	-0.494318000	-4.510300000
Ag	0.205998000	3.957563000	-5.013298000
Ag	0.691755000	1.870019000	-3.093528000
Ag	-1.946310000	2.857341000	-3.738971000
Ag	-0.843326000	-0.620055000	-3.311975000
Ag	2.329866000	2.118038000	-5.367181000

Ag_7^+-Tyr^{-1}

N	-2.619476000	-1.328704000	-0.886481000
C	-2.536032000	-0.431816000	0.267963000
C	-2.519374000	1.082642000	-0.070495000
O	-2.339098000	1.371800000	-1.304541000
H	-1.987582000	-0.958777000	-1.607675000
H	-3.551558000	-1.218759000	-1.303122000
H	-3.412841000	-0.616052000	0.911421000
C	-1.275345000	-0.740921000	1.132707000
C	0.033418000	-0.238955000	0.562677000
H	-1.244220000	-1.836667000	1.254492000
H	-1.425553000	-0.295153000	2.127510000
C	2.505837000	0.720008000	-0.494313000
C	0.724772000	0.819207000	1.184707000
C	0.612586000	-0.805030000	-0.594951000
C	1.816076000	-0.330418000	-1.129856000
C	1.952551000	1.312644000	0.671005000
H	0.329217000	1.253989000	2.107493000
H	0.118719000	-1.651136000	-1.081466000
H	2.243708000	-0.795407000	-2.023524000
H	2.557739000	2.019293000	1.250697000
O	3.708310000	1.190042000	-0.928373000
H	3.969270000	0.701377000	-1.734047000
O	-2.630858000	1.882082000	0.914622000
Ag	0.706970000	3.112850000	-0.370720000
Ag	-1.696452000	3.354175000	-2.063357000
Ag	0.802171000	4.065367000	-3.063584000
Ag	-3.063365000	5.758570000	-1.295556000
Ag	-1.873311000	4.032327000	0.663872000
Ag	-0.312865000	5.587376000	-0.958115000
Ag	-1.548364000	5.548143000	-3.674229000

Ag_7-Tyr^{-1}

N	3.651643000	-0.088933000	0.183336000
C	3.679118000	-0.872973000	1.430436000
C	2.274130000	-0.941880000	2.106130000
C	4.203799000	-2.315467000	1.233586000
C	0.579854000	-2.824161000	2.249068000
C	1.259002000	-1.869412000	1.470608000
O	4.252670000	-2.759972000	0.035546000
C	-0.389971000	-3.690229000	1.692744000
O	4.492341000	-2.975750000	2.290997000
C	0.928333000	-1.797732000	0.097260000
C	-0.688476000	-3.600998000	0.313948000
C	-0.017072000	-2.656046000	-0.481140000
O	-1.584641000	-4.430648000	-0.304928000
H	3.592682000	-0.786980000	-0.571675000
H	4.583838000	0.322177000	0.056162000
H	4.337169000	-0.364573000	2.154553000
H	2.413346000	-1.243520000	3.156419000
H	1.887732000	0.093388000	2.110233000
H	0.794543000	-2.897942000	3.320296000

H	-0.985115000	-4.344451000	2.341856000
H	1.410556000	-1.043515000	-0.529793000
H	-0.251813000	-2.592879000	-1.546935000
Ag	4.044287000	-5.229374000	1.951368000
Ag	3.310189000	-7.153134000	0.022742000
Ag	2.693481000	-4.461322000	-0.510375000
Ag	0.836018000	-6.474189000	-1.046168000
Ag	2.942459000	-7.686863000	2.719687000
Ag	0.886903000	-8.459046000	0.939930000
Ag	1.272003000	-5.741329000	1.645315000
H	-1.869629000	-5.116152000	0.332569000

Ag_8^{2+}-Tyr^{-1}

N	-2.316439000	-1.059806000	-1.973197000
C	-2.803323000	0.120201000	-1.207298000
C	-2.999914000	1.262009000	-2.235295000
O	-3.407181000	0.891745000	-3.388200000
H	-2.255893000	-1.883218000	-1.363769000
H	-3.007747000	-1.267448000	-2.701839000
H	-3.799092000	-0.099130000	-0.775236000
C	-1.868367000	0.457228000	-0.025504000
C	-0.415732000	0.682142000	-0.373889000
H	-1.942339000	-0.376600000	0.694210000
H	-2.280161000	1.349491000	0.468267000
C	2.342818000	1.086995000	-1.009360000
C	0.119105000	1.983925000	-0.499747000
C	0.471277000	-0.408911000	-0.559889000
C	1.824483000	-0.218897000	-0.888092000
C	1.484231000	2.196214000	-0.811594000
H	-0.527352000	2.847677000	-0.322928000
H	0.109982000	-1.430090000	-0.400457000
H	2.483211000	-1.082931000	-1.017953000
H	1.905292000	3.206004000	-0.810241000
O	3.644549000	1.344431000	-1.299218000
H	4.126066000	0.498892000	-1.397334000
O	-2.731151000	2.455759000	-1.875381000
Ag	0.440633000	2.338469000	-3.135712000
Ag	-0.163243000	3.374969000	-5.672391000
Ag	-1.786187000	1.171735000	-5.091742000
Ag	-2.796595000	3.304819000	-6.514651000
Ag	0.944425000	0.608125000	-5.273233000
Ag	0.356947000	5.069939000	-3.531619000
Ag	-2.119369000	3.703964000	-3.736114000
Ag	-0.444130000	-0.430579000	-3.151897000

Ag_8^{+}-Tyr^{-1}

N	2.439797000	2.129407000	-0.211901000
C	2.494222000	0.829584000	0.468753000
C	1.154006000	0.497222000	1.194997000
C	2.826524000	-0.359430000	-0.463486000
C	-0.630867000	-1.156889000	0.480933000
C	-0.007957000	0.093751000	0.310419000

O	2.780226000	-0.132819000	-1.719384000
C	-1.758784000	-1.539240000	-0.283091000
O	3.054481000	-1.481952000	0.108424000
C	-0.541702000	0.960169000	-0.669019000
C	-2.272802000	-0.639059000	-1.244292000
C	-1.644051000	0.597651000	-1.454080000
O	-3.394197000	-1.029947000	-1.944435000
H	2.154477000	1.934347000	-1.180578000
H	3.401430000	2.474189000	-0.308408000
H	3.264500000	0.874827000	1.256419000
H	1.349935000	-0.314421000	1.912486000
H	0.887638000	1.398880000	1.773639000
H	-0.263489000	-1.844220000	1.249350000
H	-2.324108000	-2.442489000	-0.030341000
H	-0.096103000	1.948744000	-0.806960000
H	-2.038339000	1.289953000	-2.204966000
H	-3.641329000	-0.321713000	-2.573131000
Ag	1.656761000	-4.294165000	-3.569611000
Ag	-2.752900000	-3.169180000	-3.331444000
Ag	1.716090000	-1.530878000	-3.145994000
Ag	2.227681000	-3.323682000	-0.918276000
Ag	-0.316158000	-2.650892000	-4.645530000
Ag	-1.004936000	-5.283819000	-3.577137000
Ag	0.661540000	-5.610307000	-1.308279000
Ag	-0.381561000	-3.045762000	-1.843694000

Ag_8-Tyr^{-1}

N	3.633308000	-0.920622000	0.017110000
C	3.535459000	-1.133160000	1.482590000
C	2.074777000	-1.054592000	1.981100000
C	4.281440000	-2.462086000	1.798337000
C	0.716331000	-3.212641000	1.988499000
C	1.097440000	-2.013635000	1.344669000
O	5.389403000	-2.618461000	1.174291000
C	-0.162141000	-4.129675000	1.375693000
O	3.753515000	-3.282286000	2.614343000
C	0.535029000	-1.747838000	0.074703000
C	-0.668219000	-3.861500000	0.088264000
C	-0.333162000	-2.656115000	-0.554759000
O	-1.478264000	-4.736163000	-0.582640000
H	4.630130000	-0.963022000	-0.223602000
H	3.291187000	0.012056000	-0.237536000
H	4.087507000	-0.334083000	2.016454000
H	2.098302000	-1.210660000	3.070486000
H	1.730496000	-0.017903000	1.810394000
H	1.092347000	-3.420326000	2.994543000
H	-0.460513000	-5.043490000	1.900126000
H	0.748243000	-0.794282000	-0.420066000
H	-0.753020000	-2.436388000	-1.539400000
H	-1.609917000	-5.531899000	-0.030575000
Ag	5.097049000	-4.413632000	-0.487778000
Ag	2.635164000	-4.814299000	0.955055000

Ag	2.812028000	-2.751711000	-1.194818000
Ag	1.727407000	-5.309178000	-1.684390000
Ag	3.690659000	-6.896015000	-0.478555000
Ag	3.953117000	-4.424766000	-3.081252000
Ag	3.118856000	-7.150669000	-3.233341000
Ag	5.742322000	-6.417709000	-2.303401000
